# Mechanistic insights into fecal microbiota transplantation for the treatment of ulcerative colitis: analysis of the STOP-Colitis trial

**DOI:** 10.1093/ecco-jcc/jjag006

**Published:** 2026-01-23

**Authors:** Mohammed Nabil Quraishi, Catherine A Moakes, Mehmet Yalchin, Claire Blackwell, Jonathan Segal, Natalie J Ives, Laura Magill, Susan E Manzoor, Konstantinos Gerasimidis, Christel McMullan, Jonathan Mathers, Richard Horniblow, Shrushma Loi, Manjinder Kaur, Nicholas J Loman, Naveen Sharma, Peter Hawkey, Victoria McCune, Joshua Quick, Samuel Nicholls, Claire McMurray, Ben Nichols, Vaios Svolos, Sebastien Raguideau, Caroline Kerbiriou, Ye H Oo, Andrew D Beggs, Nicola Crees, Richard Hansen, Ailsa L Hart, Daniel R Gaya, Christopher Quince, Tariq H Iqbal

**Affiliations:** University of Birmingham Microbiome Treatment Centre, University of Birmingham, Birmingham, United Kingdom; Department of Gastroenterology, University Hospitals Birmingham, Birmingham, United Kingdom; Department of Gastroenterology, Sheikh Shakhbout Medical City, Pure Health, Abu Dhabi, United Arab Emirates; Birmingham Clinical Trials Unit, Institute of Applied Health Research, University of Birmingham, Birmingham, United Kingdom; Department of Gastroenterology, St Mark’s Hospital, London, United Kingdom; Gastroenterology Unit, Glasgow Royal Infirmary, Glasgow, United Kingdom; Department of Gastroenterology, St Mark’s Hospital, London, United Kingdom; Birmingham Clinical Trials Unit, Institute of Applied Health Research, University of Birmingham, Birmingham, United Kingdom; Birmingham Clinical Trials Unit, Institute of Applied Health Research, University of Birmingham, Birmingham, United Kingdom; University of Birmingham Microbiome Treatment Centre, University of Birmingham, Birmingham, United Kingdom; Human Nutrition, University of Glasgow, Glasgow, United Kingdom; Institute of Applied Health Research, University of Birmingham, Birmingham, United Kingdom; Institute of Applied Health Research, University of Birmingham, Birmingham, United Kingdom; University of Birmingham Microbiome Treatment Centre, University of Birmingham, Birmingham, United Kingdom; Birmingham Clinical Trials Unit, Institute of Applied Health Research, University of Birmingham, Birmingham, United Kingdom; Birmingham Clinical Trials Unit, Institute of Applied Health Research, University of Birmingham, Birmingham, United Kingdom; Institute of Microbiology and Infection, University of Birmingham, Birmingham, United Kingdom; University of Birmingham Microbiome Treatment Centre, University of Birmingham, Birmingham, United Kingdom; Department of Gastroenterology, University Hospitals Birmingham, Birmingham, United Kingdom; University of Birmingham Microbiome Treatment Centre, University of Birmingham, Birmingham, United Kingdom; South Tees NHS Foundation Trust, Middlesbrough, United Kingdom; Institute of Microbiology and Infection, University of Birmingham, Birmingham, United Kingdom; Institute of Microbiology and Infection, University of Birmingham, Birmingham, United Kingdom; Institute of Microbiology and Infection, University of Birmingham, Birmingham, United Kingdom; Human Nutrition, University of Glasgow, Glasgow, United Kingdom; Human Nutrition, University of Glasgow, Glasgow, United Kingdom; Organisms and Ecosystems, Earlham Institute, Norwich, United Kingdom; Human Nutrition, University of Glasgow, Glasgow, United Kingdom; Department of Gastroenterology, University Hospitals Birmingham, Birmingham, United Kingdom; National Institute for Health and Care Research (NIHR) Birmingham Biomedical Research Centre, Centre for Liver and Gastrointestinal Research, Birmingham, United Kingdom; University of Birmingham Microbiome Treatment Centre, University of Birmingham, Birmingham, United Kingdom; National Institute for Health and Care Research (NIHR) Birmingham Biomedical Research Centre, Centre for Liver and Gastrointestinal Research, Birmingham, United Kingdom; Crohn’s and Colitis UK, United Kingdom; School of Medicine, University of Dundee, Dundee, United Kingdom; Department of Gastroenterology, St Mark’s Hospital, London, United Kingdom; Gastroenterology Unit, Glasgow Royal Infirmary, Glasgow, United Kingdom; Organisms and Ecosystems, Earlham Institute, Norwich, United Kingdom; University of Birmingham Microbiome Treatment Centre, University of Birmingham, Birmingham, United Kingdom; Department of Gastroenterology, University Hospitals Birmingham, Birmingham, United Kingdom; National Institute for Health and Care Research (NIHR) Birmingham Biomedical Research Centre, Centre for Liver and Gastrointestinal Research, Birmingham, United Kingdom

**Keywords:** fecal microbiota transplantation, ulcerative colitis, immunology

## Abstract

**Background and Aims:**

Fecal microbiota transplantation (FMT) is a promising therapy for ulcerative colitis, but variable responses and unclear mechanisms limit its efficacy. We aimed to compare nasogastric versus colonic FMT delivery and define the microbial and immunological changes associated with clinical response.

**Methods:**

In this prospective, open-label, randomized pilot trial (STOP-Colitis), 30 adults with active ulcerative colitis were randomized to receive multidose FMT via nasogastric tube or colonoscopy with subsequent enemas. Key endpoints were clinical outcomes at week 8 and longitudinal multi-omic analyses of stool and biopsies to define changes in microbial composition (16S rRNA and shotgun metagenomics), short-chain fatty acids (SCFAs), mucosal T-cells, and host gene expression.

**Results:**

Colonic FMT was superior to nasogastric delivery, with a higher clinical response rate at week 8 (75% [9/12] vs 25% [2/8]; risk ratio 2.94, 95% CI 0.84-10.30—per protocol analysis). Response was underpinned by successful microbial engraftment, leading to significantly increased fecal microbial diversity and enrichment of SCFA-producing taxa, including Oscillospiraceae and Christensenellaceae. This correlated with reduced fecal calprotectin. Responders showed a significant increase in mucosal regulatory T cells (*P* = .01), with a concurrent decrease in Th17 (*P* = 0.03) and CD8+ T cells. This anti-inflammatory shift was confirmed by mucosal transcriptomics, which revealed upregulation of metabolic pathways and downregulation of proinflammatory defense pathways in responders. (Trial registration: ISRCTN74072945).

**Conclusion:**

Colonic FMT is a more effective delivery route than nasogastric administration. Clinical response is driven by the engraftment of immunomodulatory bacteria that restore a healthy host–microbe dialogue, providing rationale for developing targeted microbial therapeutics.

## 1. Introduction

Ulcerative colitis (UC) is a chronic inflammatory bowel disease (IBD) characterized by persistent inflammation of the colonic mucosa, often presenting with symptoms such as bloody diarrhea and abdominal pain.^1^^,^^2^ Although the precise cause of UC remains uncertain, it is thought to result from a dysregulated immune response to gut microbiota in genetically predisposed individuals under the influence of unknown environmental factors.^3–8^ Increasing evidence suggests that an imbalance in the gut microbiota plays a key role in UC by driving inappropriate immune responses and chronic inflammation. In UC, changes in the gut microbiota, such as reductions in both diversity and certain obligatory anaerobic “beneficial” species, along with increased pathobionts have been shown to disrupt immune balance and lead to excessive inflammation.^9–11^ Fecal microbiota transplantation (FMT) offers a promising therapeutic strategy to correct dysbiosis by restoring microbial diversity and composition and potentially re-establishing immune balance.

Eight double-blind, randomized, placebo-controlled trials (RCTs) have investigated FMT for UC, with six showing positive outcomes in achieving clinical and endoscopic remission.^12–19^ However, variations in FMT preparation, donor selection, and delivery methods pose challenges for translating these findings into clinical practice.^20–22^ Additionally, the lack of detailed immunological analysis makes it difficult to fully understand how microbial shifts or the introduction of donor microbial strains contribute to remission, particularly important when this is not sustained in the long term.

The STOP-Colitis pilot trial evaluated two possible FMT delivery routes in patients with active UC, nasogastric and colonic administration, via an RCT. This prospective, open-label, multicenter study recruited 30 patients from three hospitals in the UK, aiming to determine the most appropriate delivery route for a future full-scale RCT and assess feasibility. Key endpoints included clinical response, acceptability, and safety, with follow-up at 8 and 12 weeks. Mechanistic outcomes, such as changes in the microbiome and inflammatory markers, were also assessed to better understand the therapeutic mechanisms of FMT.

The primary outcome of the pilot was a composite qualitative assessment to assess the best route of FMT delivery to take forward to an efficacy-powered RCT. This paper presents biological mechanistic findings from the STOP-Colitis trial, demonstrating how colonic FMT induces specific microbial shifts and immune modulation, which are crucial for understanding its therapeutic effects and for guiding the development of future targeted microbial interventions in UC.

## 2. Materials and methods

### 2.1. Study design and participants

The STOP-Colitis trial was a prospective, multicenter, open-label, randomized pilot trial conducted at three UK hospitals to compare nasogastric versus colonic FMT for active UC. The full trial protocol has been published.^23^ Eligible participants were adults (aged 16–70 years) with active UC, defined by a Partial Mayo Score of 4-8, including a rectal bleeding subscore of at least 1. Participants were required to be on stable doses of maintenance therapy (eg, 5-aminosalicylates or immunomodulators) or on no treatment. Key exclusion criteria included active infections, pregnancy, and recent use (within protocol-defined washout periods) of antibiotics, biologics, JAK inhibitors, or corticosteroids. All participants provided written informed consent before any study procedures. The trial was conducted in accordance with the *Declaration of Helsinki* and Good Clinical Practice guidelines, received ethical approval from the East Midlands-Nottingham Research Ethics Committee (17/EM/0274), and was registered (ISRCTN74072945). The full methods are available as Supplementary Material.

### 2.2. Randomization and masking

Using a central computer-generated system, participants were randomized 1:1 to either the nasogastric or colonic FMT delivery arm. Randomization was stratified by baseline partial Mayo score (4-5 or 6-8) and smoking status (current vs non-smoker). Given the distinct nature of the delivery methods, the trial was open-label for both participants and investigators.

### 2.3. Procedures

FMT material was produced at the University of Birmingham Microbiome Treatment Centre under a Good Manufacturing Practices specials license (MHRA MS 21761). Stool was sourced from rigorously screened healthy volunteer donors, processed under anaerobic conditions, and cryopreserved at –80 °C.

Participants in the nasogastric group received four daily 50-mL infusions of FMT (approx. 30 g stool equivalent per infusion) via a nasogastric tube at week 0 and again at week 4. Participants in the colonic group received a single 250-mL FMT administration (approx. 150 g stool equivalent) via colonoscopy to the cecum at week 0, followed by seven weekly 100-mL self-administered enemas (approx. 30 g stool equivalent per enema). Prior to the first FMT administration, all participants underwent bowel preparation with a macrogol-based solution. To promote retention, loperamide (4 mg) was administered orally before and after each infusion.

### 2.4. Mechanistic assessments

Fecal samples, colonic mucosal biopsies, and whole blood were collected at baseline and specified follow-up points. For microbiome analysis, DNA was extracted from fecal and mucosal samples for 16S rRNA gene (V4 region) and shotgun metagenomic sequencing on an Illumina NovaSeq platform. Analyses focused on changes in microbial diversity, taxonomic composition, and functional potential, including the assembly of metagenome-assembled genomes (MAGs). For metabolomics, fecal short-chain fatty acids (SCFAs), including acetate, propionate, and butyrate, were quantified using gas chromatography. For immunological analysis, lamina propria mononuclear cells were isolated from fresh colonic biopsies, and peripheral blood mononuclear cells were isolated from whole blood. Cell populations, including regulatory T cells (Tregs), T-helper 17 (Th17) cells, and their functional subsets, were phenotyped using multiparameter flow cytometry. Intracellular cytokine production was assessed following *ex vivo* stimulation. For transcriptomics, total RNA was extracted from colonic biopsies (RNA integrity number [RIN] ≥  7.0) for bulk RNA sequencing to evaluate differential gene expression and pathway modulation in response to FMT. Detailed laboratory and bioinformatic protocols for all mechanistic analyses are provided in the supplementary appendix. Finally the habitual dietary pattern of the donors was assessed at the time of first donation using the validated EPIC-Norfolk food frequency questionnaire.^24^ Data were analyzed to estimate energy, macronutrient, and fiber intake to explore correlations between donor diet and donor microbiome characteristics.

### 2.5. Outcomes

The primary outcome of this pilot trial was a composite qualitative assessment of feasibility and tolerability to determine the optimal delivery route for a future definitive efficacy trial. Key secondary clinical outcomes reported here include clinical response (a decrease of ≥3 points and ≥30% from baseline in the partial Mayo score), clinical remission (partial Mayo score ≤2 with no individual sub-score >1), and mucosal healing (endoscopic Mayo score of 0 or 1) at week 8. Mechanistic outcomes included the longitudinal changes in the microbial, metabolic, immunological, and transcriptomic profiles described above. Assessments were performed at baseline and at weeks 2, 4, 6, 8, and 12.

### 2.6. Statistical analysis

As a pilot and feasibility study, a formal sample size calculation for efficacy was not performed. All clinical outcomes were analyzed on an intention-to-treat basis, including all randomized participants who received at least one dose of FMT. Risk ratios (RRs) and 95% confidence intervals (CIs) for binary clinical outcomes were calculated using a log-binomial regression model, adjusting for stratification variables. Missing data for binary endpoints were imputed as no response. Changes in microbial alpha diversity and immune cell frequencies over time were assessed using paired *t*-tests or Wilcoxon signed-rank tests. Overall microbial community composition (beta diversity) was analyzed with permutational multivariate analysis of variance (PERMANOVA). Differential abundance of taxa and correlations between microbial and immunological markers were assessed using appropriate non-parametric tests with Benjamini–Hochberg correction for multiple comparisons. A repeated-measures mixed-effects model was used to analyze changes in SCFAs over time. For transcriptomics, differential gene expression was assessed using a quasi-likelihood F-test. A *P*-value or false discovery rate (FDR)-adjusted *P*-value of<.05 was considered statistically significant. Further details on statistical methods are available in the supplementary appendix.

## 3. Results

### 3.1. Colonic administration of FMT demonstrates superior clinical outcomes and adherence compared to nasogastric delivery in UC patients

Seventy-six participants with active UC were screened, with 37 registered and 30 randomized: 16 to the nasogastric arm and 14 to the colonic arm (Figure S1). Seven participants in the nasogastric arm and two in the colonic arm withdrew from follow-up. An additional participant in the nasogastric arm did not attend the 8-week assessment, and thus primary clinical outcome data were available for eight participants in this arm. Baseline demographics and disease activity are shown in Table 1. Inflammation indices were generally balanced between groups, though participants in the nasogastric arm were younger and more recently diagnosed.

**Table 1.** Patient demographics and disease characteristics.

	FMT naso-gastric route (*N* = 16)	FMT colonic route (*N* = 14)
**Age at randomization (years), mean (SD, *N*)**	37.3 (11.0, 16)	46.1 (11.7, 14)
**Gender, *n* (%)**		
** Male**	9 (56)	5 (36)
**Female**	7 (44)	9 (64)
**Partial Mayo score^a,b^, *n* (%)**		
** 4-5**	5 (31)	5 (36)
** 6-8**	11 (69)	9 (64)
**Full Mayo score^c^, mean (SD, *N*)**	7.8 (1.3, 16)	8.1 (1.5, 14)
**Current smoker^b,d^, *n* (%)**	2 (13)	2 (14)
**Duration of diagnosis of UC (years)^e^, median [IQR, *N*]**	4.4 [1.5-6.4, 16]	8.5 [3.0-12.0, 14]
**Disease extent, *n* (%)**		
** Left-sided disease**	9 (56)	9 (64)
** Pancolitis**	7 (44)	3 (22)
** Proctitis**	0 (-)	2 (14)
**Previous biologics used, *n* (%)**		
** Infliximab**	3 (19)	3 (21)
** Vedolizumab**	4 (25)	2 (14)
** Adalimumab**	1 (6)	0 (-)
** Golimumab**	1 (6)	1 (7)
**Taking maintenance therapy for ulcerative colitis^f^, *n* (%)**	13 (81)	13 (93)
** Oral 5ASA compound**	10	12
** Immunosuppressants^g^**	5	3
**Other concomitant medications**	5^h^	7^i^
**Hemoglobin (g/L), mean (SD, *N*)**	126.0 (18.9, 16)	129.6 (17.1, 14)
**Albumin (g/L), mean (SD, *N*)**	41.9 (5.3, 15)	43.5 (3.9, 13)
**C-reactive protein (mg/L), mean (SD, *N*)**	11.3 (13.0, 16)	4.2 (3.4, 14)
**Fecal calprotectin ([mg/kg]^b^), mean (SD, *N*)**	670.8 (582.6, 11)	750.7 (343.7, 11)

a Stratification variable for randomization.

b Range 0-9, with higher scores indicating more severe disease.

c Range 0-12, combining clinical and endoscopic assessments, with higher scores indicating more severe disease.

d Stratification variable for randomization.

e Time from initial UC diagnosis to trial enrolment.

f Patients receiving ongoing UC medications at baseline, excluding corticosteroids.

g Including azathioprine, mercaptopurine, or methotrexate.

h Number of concomitant medications in nasogastric arm.

i Number of concomitant medications in colonic arm.

Clinical response was observed in 75% (9/12, 95% CI 51-100%) of the colonic arm and 25% (2/8, 95% CI 0-55%) of the nasogastric arm (RR 2.94, 95% CI 0.84-10.30). Clinical remission was also more frequent in the colonic arm (6/12 vs 2/8; RR 1.89, 95% CI 0.51-6.99). Partial Mayo clinical response was reported in 86% (12/14) of the colonic arm and 56% (9/16) of the nasogastric arm (hazard ratio [HR] 1.16, 95% CI 0.48-2.80). Mucosal healing rates were 25% in both arms (3/12 in the colonic arm and 2/8 in the nasogastric arm). Weight and quality of life scores at weeks 8 and 12 were similar between groups.

Adverse events (AEs) occurred in 69% (11/16) of participants in the nasogastric arm and 79% (11/14) in the colonic arm. The most frequently reported AEs in the nasogastric arm were abdominal pain (*n* = 7) and nausea (*n* = 5), while the colonic arm most frequently reported diarrhea (*n* = 10) and abdominal pain (*n* = 4). However, serious adverse events (SAEs) were reported only in the nasogastric arm, where two participants were hospitalized due to abdominal pain, fever, and gastrointestinal symptoms, leading to withdrawal from follow-up. No SAEs were recorded in the colonic arm.

### 3.2. Donor microbiome characteristics show limited association with FMT response

**Figure 1.**
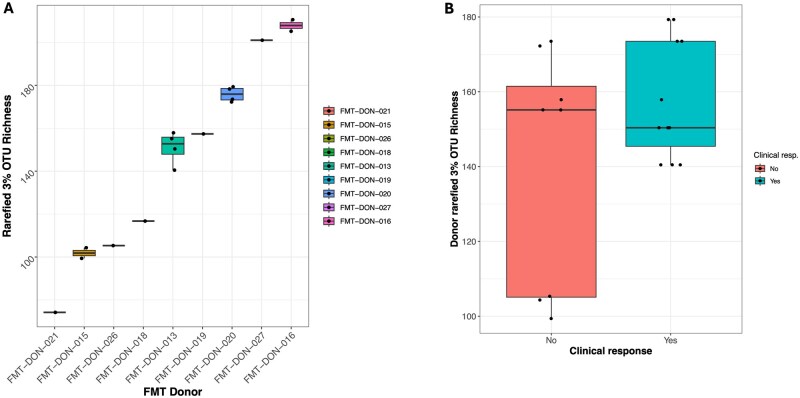
Donor microbiome diversity shows limited association with fecal microbiota transplantation (FMT) response. (A) Boxplot of donor microbiome diversity, measured by rarefied 16S rRNA gene 3% operational taxonomic unit (OTU) number across all donor samples. Data are represented as median with interquartile range. (B) Comparison of donor microbiome diversity for recipients who achieved clinical response (*n* = 11) versus non-responders (*n* = 8). Data are represented as median with interquartile range. Statistical significance was determined by a two-sample *t*-test.

We used 16S rRNA gene sequencing and shotgun metagenomics to characterize the microbiome of both recipients over time and donors. We obtained sufficient 16S rRNA reads (>10 000) for analysis from 161 samples in total, with a median of 52 780 reads per sample. Across donor–recipient pairs (*N* = 19) we observed a non-significantly higher donor microbiome diversity for recipients who responded (*N* = 11) to the FMT, based on rarefied 16S rRNA gene 3% operational taxonomic unit (OTU) number (*t*-test mean difference = 17.4, *P* = .18—see Figure 1). Analysis of OTUs with non-zero abundance in at least 10 donor samples (*N* = 156) identified several OTUs potentially associated with response. Notably, an OTU from the family Peptostreptococcaceae demonstrated a significant negative association with response (adjusted *P*-value <.1—see Table S1).

Donor metagenome MetaPhlAn4 species diversity from the shotgun metagenomes showed no significant association with response (*P* = .51), a finding that extended to MAGs and donor metagenome community composition.

Analysis of donor habitual dietary intake, assessed by a food frequency questionnaire (*N* = 9 donors), found no significant correlations between energy, macronutrient, or fiber intake and donor microbiome diversity. A moderate, non-significant positive correlation was observed between the intake of milk and milk products and rarefied species (Pearson’s *r* = .67), but overall, donor dietary patterns were not found to be associated with recipient clinical response in this cohort.

### 3.3. Increased microbiome diversity observed in colonic FMT recipients and responders

Fecal microbiome diversity—as measured by 16S rRNA gene 3% OTU numbers—increased significantly from baseline to day 56 across all FMT recipients (*N* = 17, mean difference = −25.0, *P* = .0064). When stratified by response, the increase in diversity was evident in both responders (*N* = 9, mean difference = −26.5, *P* = .06) and non-responders (*N* = 8, mean difference = −23.43, *P* = .07).

**Figure 2.**
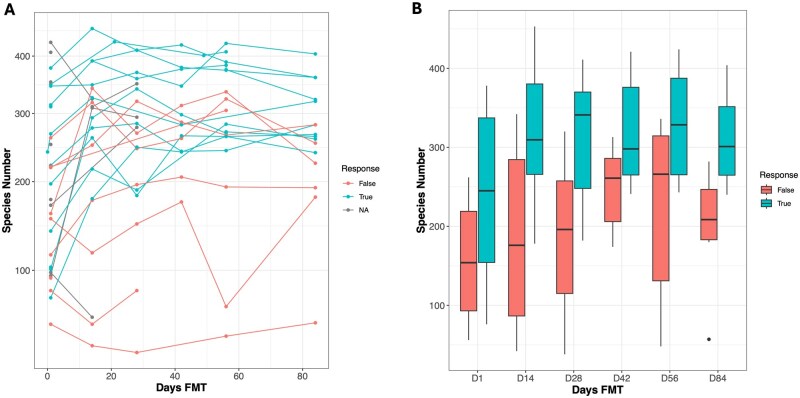
Microbiome diversity increases in responders following fecal microbiota transplantation (FMT). (A) Trajectory of recipient microbiome species richness for each patient (*n* = 16 biological replicates) over the 56-day treatment period, colored by clinical response status. (B) Boxplots of recipient species richness at indicated time points, comparing responders (*n* = 9) and non-responders (*n* = 7). Data are represented as median with interquartile range. Statistical significance was determined by a two-sample *t*-test. *P < .05.

Shotgun metagenomic data revealed more pronounced trends, with a significant increase in species richness across all patients (*N* = 16, mean difference = −72.625, *P* = .001). Responders exhibited a robust increase in species richness (*N* = 9, *P* = .0027), whereas non-responders showed a non-significant change (*N* = 7, *P* = .1498). Notably, while the largest increase in species richness occurred by day 56, a significant rise was already detectable by day 14, indicating an early impact of the intervention (Figure 2A and Table S2). Note that these changes resulted in a difference in diversity between the responder and non-responder groups (see Figure 2B) with a significantly lower diversity in the latter at day 28 (*t*-test *N* = 16, *P* = .02), even at day 1 there was a significantly lower diversity in non-responders (*N* = 19, *P* = .05).

### 3.4. Recipient microbiome convergence with donor profiles is more pronounced in FMT responders

**Figure 3.**
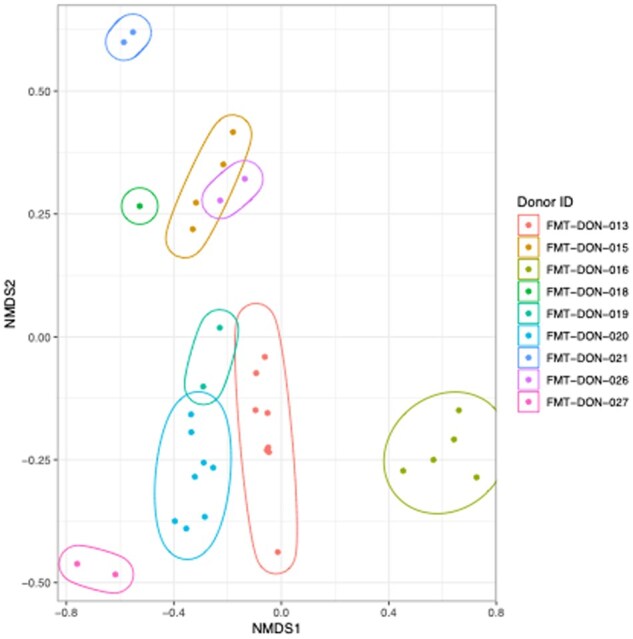
Donor microbiome composition is highly variable. Non-metric multidimensional scaling (NMDS) plot illustrating the composition of donor microbiomes based on Bray–Curtis distances of MetaPhlAn4 species profiles. Each point represents a sample from a unique donor. Statistical significance was determined by PERMANOVA (*R*^2^ = .83, *P* < .001).

**Figure 4.**
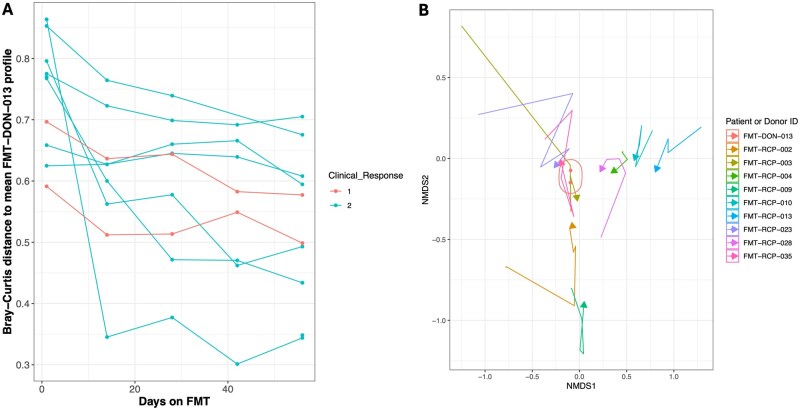
Recipient microbiome convergence toward a donor profile is more pronounced in responders. (A) Bray–Curtis distance between recipient and donor (FMT-DON-013) microbiomes over time for responders and non-responders. Data are represented as mean ± SEM. Statistical analysis by Pearson’s correlation. (B) Non-metric multidimensional scaling (NMDS) plot illustrating the convergence of recipient fecal microbiota (*n* = 9 biological replicates) toward the donor profile (FMT-DON-013) from day 1 to day 56. Arrows indicate the trajectory for each patient.

Donor microbiome composition, analyzed using MetaPhlAn4 species profiles, showed significant variability (multivariate permutation ANOVA with Bray–Curtis distances: *R*^2^ = .83, *P* < .001; Figure 3). During the FMT process, recipient fecal microbiomes progressively converged toward their respective donor profiles, with this trajectory becoming evident by day 56 (illustrated using the most frequently used donor [FMT-DON-013] in Figure 4). The Bray–Curtis distance between recipient and donor microbiomes showed a strong negative correlation with the duration of FMT treatment (Pearson’s correlation, *N* = 104, *r* = −.44, *P* < .001), reflecting increasing similarity over time. This correlation was stronger in responders (*N* = 54, *r* = −.47, *P* < .001) than in non-responders (*N* = 33, *r* = −.36, *P* = .039). Using 3% 16S rRNA gene profiles, a similar but weaker pattern was observed, with correlations of *r* = −.27 (*P* = .03) in responders and *r* = −.03 (*P* = .80) in non-responders. These results indicate differential microbiome adaptation dynamics, with responders demonstrating more consistent and pronounced donor–recipient microbiome convergence compared to non-responders.

### 3.5. Distinct microbiome shifts and microbial engraftment occur predominantly in FMT responders

From day 1 to day 56, significant changes in microbiome composition were observed across all FMT recipients (*N* = 17), with 15 16S rRNA 3% OTUs showing increased abundance (paired *t*-test Benjamini–Hochberg FDR < .10), including taxa from families such as Christensenellaceae, Oscillospiraceae, and one Bacteroides species (Table S3). When restricted to responders (*N* = 9), only OTU_51 from Christensenellaceae demonstrated significant changes, while no significant changes were observed in the non-responders (*N* = 8). Metagenomic species profiling via MetaPhlAn4 revealed more significant changes reflecting the higher resolution of this approach over 16S rRNA gene sequencing. Across all patients (*N* = 16), 78 species significantly changed in abundance (FDR < .10), with 77 species increasing and *Ruminococcus torques* being the sole species to decrease (Table S4).

**Figure 5.**
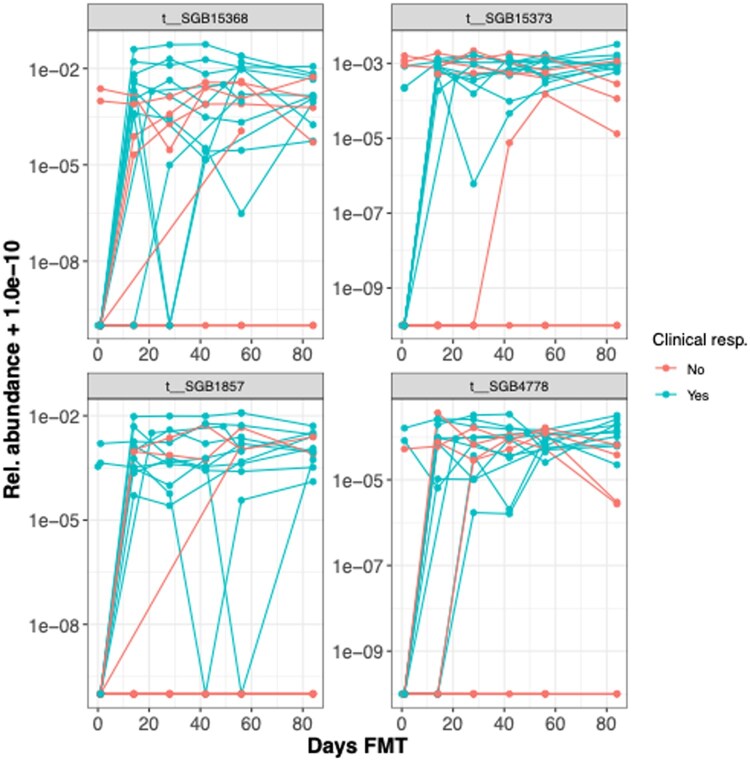
Specific microbial species are enriched in responders following fecal microbiota transplantation (FMT). Relative abundance of the four most significantly changing microbial species from day 1 to day 56 in the responder group (*n* = 9 biological replicates). Each line connects measurements from a single patient. The *y*-axis is log_10_ scaled. Statistical significance was determined by a paired *t*-test with Benjamini–Hochberg FDR correction. See also Table S5.

In responders, 24 species showed significant increases in abundance (Table S5), predominantly from the phylum Firmicutes (15/24) and family Oscillospiraceae (5/24). Notably, six species were from the phylum Bacteroidetes. The presence of specific species at day 56, such as *Firmicutes t__SGB15368* (absent at day 1), indicated microbial engraftment unique to responders. Figure 5 illustrates the relative abundance trajectories of some key species in responders, highlighting this pattern, which was absent in non-responders.

Six *de novo* reconstructed microbial genomes (dMAGs) exhibited significant changes in responders, including two strains of *Mediterraneibacter butyricigenes* (Table S6). Conversely, no significant changes were detected in non-responders for either species or dMAGs during FMT. These findings emphasize that substantial microbial engraftment and compositional shifts are confined to the responder group, underscoring differential microbiome dynamics based on clinical outcomes.

Analysis of community-level functional potential showed no significant changes in KEGG (Kyoto Encyclopedia of Genes and Genomes) ortholog abundance following FMT between day 1 and day 56, after adjustment for multiple comparisons, suggesting that clinical effects were driven by species-specific rather than aggregate functional shifts.

### 3.6. Lower fecal calprotectin levels in FMT responders correlate with increased microbial diversity

**Figure 6.**
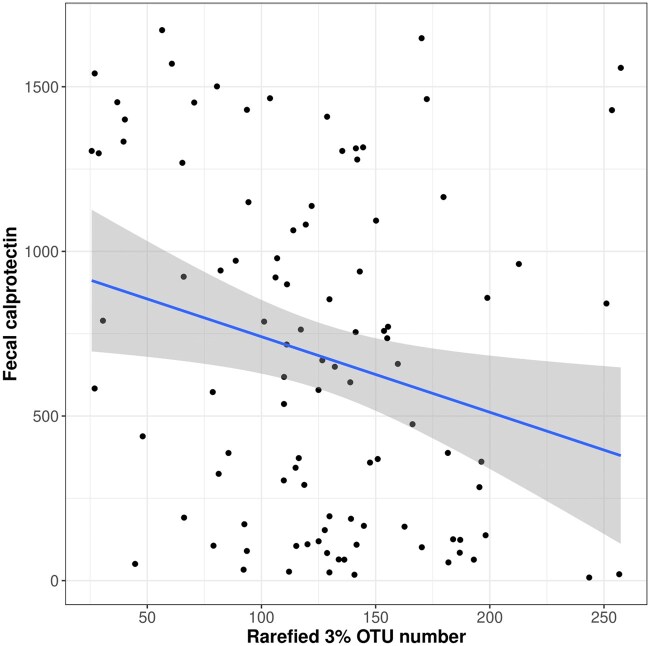
Fecal calprotectin levels correlate negatively with microbial diversity. Correlation of fecal calprotectin (μg/g) with rarefied 16S rRNA gene 3% operational taxonomic unit (out) richness across all patients and time points (*n* = 99 samples from 19 patients). The solid line indicates the best fit from a linear regression. Statistical significance was determined by Kendall’s tau correlation test.

Responders to FMT exhibited significantly lower fecal calprotectin levels compared to non-responders during weeks 4-8; comparing average calprotectin levels over these times we observed a difference between the two groups (responders mean 509 μg/g vs non-responders 854 μg/g, respectively; *P* = .03; Figure 6). Across all time points, higher fecal calprotectin levels were negatively correlated with reduced microbial alpha diversity, as indicated by the rarefied 3% OTU number (*N* = 99, tau = −0.18; *P* = .00949) (Table S7). This correlation was strongest on day 28, with an increased negative association observed (*N* = 17, tau = −0.47; *P* = .007921). Similarly, MetaPhlAn4 species analysis revealed a significant negative association between species number and fecal calprotectin across all time points (*N* = 99, tau = −0.18; *P* = .008014), with a stronger association on day 28 (*N* = 17, tau = −0.46; *P* = .009395).

### 3.7. SCFA concentrations increase during FMT but are not associated with clinical response

We observed a significant increase in fecal concentrations of key SCFAs over the course of FMT. Across all participants with available data (*N* = 19), the concentration of acetate increased by 45% from baseline to week 2 (*P* = .05), and butyrate increased by 57% over the same period (*P* = .03). These increases persisted over the full treatment period in participants with paired data at week 8 (*N* = 16), with acetate showing a 21% increase (*P* = .02) and butyrate a 39% increase (*P* = .08) compared to baseline. No significant changes were observed for propionate (2 weeks: *P* = .46; 8 weeks: *P* = .36) or other SCFAs measured.

In repeated-measures modeling adjusted for treatment allocation and clinical response, there was a consistent effect of time across all measured metabolites, but no significant time-by-treatment or time-by-response interactions. Notably, when analyses were restricted to participants who achieved clinical response (*N* = 9), the increases in acetate and butyrate observed at weeks 2 and 8 were no longer statistically significant, although trends toward elevation remained.

### 3.8. Selective modulation of mucosal T cell subsets following FMT: increased regulatory and IL-10-producing T cells with reduced Th17 and CD8 T cells

**Figure 7.**
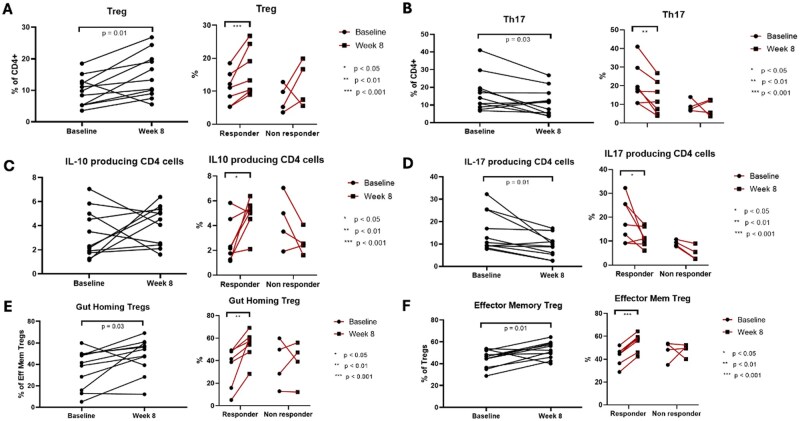
Fecal microbiota transplantation (FMT) induces a selective anti-inflammatory modulation of mucosal T cell subsets in responders. Change in frequency of specific T cell populations in the colonic mucosa from baseline to week 8 for the entire cohort (*n* = 11 biological replicates), responders (*n* = 7), and non-responders (*n* = 4). (A) Regulatory T cells (Tregs). (B) Th17 cells. (C) IL-10 producing CD4+ T cells. (D) IL-17 producing CD4+ T cells. (E) Gut-homing effector memory Tregs. (F) Effector memory Tregs. Data are represented as mean ± SEM. Statistical significance was determined by a paired *t*-test. *P < .05, **P < .01, ***P < .001.

In the cohort with paired biopsies available for analysis (*N* = 11, comprising seven responders and four non-responders), FMT was associated with an increase in colonic mucosal regulatory T cells and a decrease in Th17 and CD8 T cells, with the frequency of regulatory T cells (CD127-CD25+FoxP3+) showing a statistically significant increase at week 8 compared to baseline (Δ = 5.02%; *P* = .01), as demonstrated in Figure 7A. This increase was more pronounced in responders (Δ = 5.29%; *P* = .009) than in non-responders (Δ = 4.55%; *P* = .36). Similarly, the frequency of Th17 cells (CCR+CD161+) significantly decreased at week 8 (Δ = −5.13%; *P* = .03), with a greater reduction in responders (Δ = −7.61%; *P* = .017) compared to non-responders (Δ = −0.78%; *P* = .83) as shown in Figure 7B. CD8 T cells also exhibited a significant decrease (Δ = −4.41%; *P* = .01), again with a more substantial reduction in responders (Δ = −5.18%; *P* = .04) than in non-responders (Δ = −3.06%; *P* = 0.22). FMT was also associated with an increase in colonic mucosal IL-10-producing CD4 T cells and a decrease in IL-17-producing CD4 T cells, consistent with an increase in regulatory T cells. Responders experienced a significant increase in IL-10-producing CD4 T cells at week 8 compared to baseline (Δ = 2.16%; *P* = .04; Figure 7C), while non-responders exhibited a non-significant reduction (Δ = −1.71%; *P* = .16). The frequency of IL-17-producing CD4 T cells decreased significantly at week 8 (Δ = −6.41%; *P* = .01), with a more pronounced reduction in responders (Δ = −7.69%; *P* = .05) compared to non-responders (Δ = −4.17%; *P* = .07; Figure 7D). No significant changes were observed in the frequencies of Th1-like CD4 cells (CCR6-CD161-CXCR3+CCR5+), Th2-like CD4 cells (CCR6-CD161-CXCR3-CCR5-), or B cells (CD45+CD19+), nor were there significant differences in the levels of IFNγ, TNFα, IL-5, IL-13, or dual IFNγ/IL-17-producing CD4 T cells after treatment. These findings highlight a selective modulation of T cell subsets, particularly in responders, following FMT.

The frequencies of mucosal gut-homing effector memory regulatory T cells (CD14-CD19-CD45+CD3+CD8-CD4+CD25+CD127-CCR7-CD45RA-alpha4+) showed a statistically significant increase at week 8 compared to baseline (Δ = 12.12%; *P* = .03, Figure 7E). Subgroup analysis based on clinical outcomes revealed that this increase was both significant and more pronounced in responders (Δ = 18.55%; *P* = .008) compared to non-responders (Δ = 0.86%; *P* = .92). Similarly, the frequencies of effector memory regulatory T cells also demonstrated a statistically significant increase at week 8 compared to baseline (Δ = 7.86%; *P* = .01; Figure 7F). Subgroup analysis indicated that this increase was significantly greater in responders (Δ = 12%; *P* <.0001) compared to non-responders (Δ = 0.63%; *P* = .92).

In the cohort with paired peripheral blood mononuclear cells available for analysis (*N* = 12, comprising eight responders and four non-responders), peripheral IL-10-producing CD4 T cell subsets showed a significant increase in responders following FMT (Δ = 0.45%; *P* = .02), a change that was not observed in non-responders or the cohort as a whole. No significant differences were found in the frequencies of IFNγ, TNFα, IL-17, IL-5, IL-13, or dual-producing IFNγ/IL-17 peripheral CD4 T cells following FMT. Additionally, the frequencies of peripheral Tregs (including Treg subsets), Th17, Th1-like, Th2-like, CD8 T cells, and B cells did not demonstrate significant changes at week 8 compared to baseline, regardless of clinical outcome.

### 3.9. Mucosal gut-homing Tregs correlate positively with Firmicutes and Verrucomicrobiota OTUs, while Th17 cells show a negative association with an Actinomycetota strain

**Figure 8.**
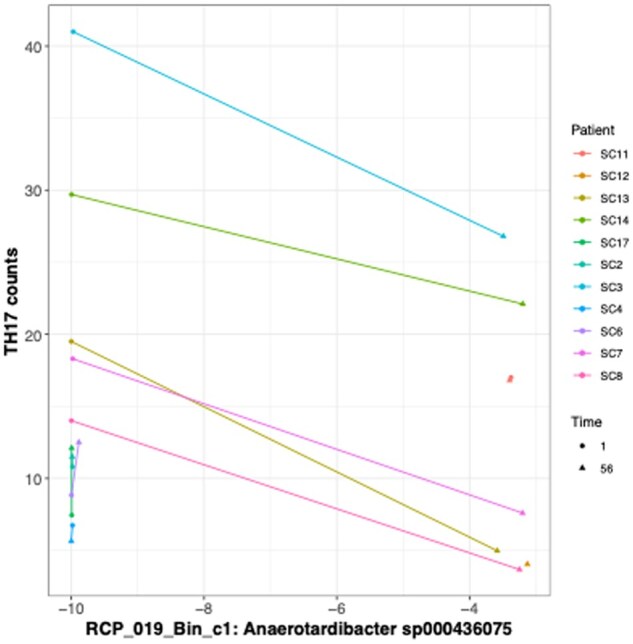
A specific *de novo* reconstructed microbial genome (dMAG) is negatively associated with Th17 cell frequency. Correlation between the log-transformed relative abundance of dMAG RCP_019_Bin_c1 *Anaerotardibacter sp000436075* and the frequency of Th17 cells in the colonic mucosa. Each line connects paired measurements from a single patient (*n* = 11 biological replicates). Statistical significance was determined by Spearman’s rank correlation.

In a subset of patients (*N* = 11) with mucosal immune subset analysis and 16S rRNA gene sequencing data, several OTUs were significantly correlated (adjusted *P*-value < .1) with changes in gut-homing Treg abundance after correcting for multiple comparisons (see Table S8). The strongest association was observed with an OTU from the genus *Victivallis* in the phylum Verrucomicrobiota, followed by two OTUs from the Firmicutes, originating from the genera *Eubacterium* and *Ruminococcus*. All these associations were positively correlated with gut-homing Tregs. No other significant associations were identified between immune cell data and OTUs after adjustment (adjusted *P*-value < .10), though taxonomically similar OTUs were near significance in their negative association with CD8 cell abundance (adjusted *P*-value = .12). For MetaPhlAn4 species-level analysis, no significant associations with immune cell counts were detected. However, one dMAG (RCP_019_Bin_c1) was significantly negatively associated with Th17 cell counts (adjusted *P*-value = .0138). This dMAG was identified as *Anaerotardibacter sp000436075* from the phylum Actinomycetota and family Eggerthellaceae (Figure 8).

### 3.10. Changes in mucosal microbiota are associated with FMT but not with response

We obtained sufficient 16S rRNA reads for analysis (>10 000) from a total of 111 mucosal biopsy samples derived from 29 individuals. Mucosal community diversity as measured by either rarefied 3% OTU number or Shannon diversity was not impacted by treatment. In contrast, we found, using permutation ANOVA with Bray–Curtis distances, that 3.3% of community composition was explained by pre- or post-treatment (*P* < .001) with subject explaining 64% (*P* < .001). The biopsy location (*P* = 0.303) was not significant. In 18 individuals with paired samples we found 21 OTUs that were significantly associated with treatment (see Table S9). The majority of OTUs were increasing with treatment, and these derived from a variety of commensal microbiota principally Lachnospiraceae^7^ but also Bacteroidaceae^2^ and Ruminococcaceae^2^; however, two OTUs decreased including one OTU_24 that was assigned to *Sutterella wadsworthensis.* When restricted to the responder group (*n* = 45) only OTU_18 a Lachnospiraceae remained significant (FDR-corrected *P* value = .001).

### 3.11. FMT response is marked by metabolic reprogramming and suppression of antimicrobial defence pathways in responders

**Figure 9.**
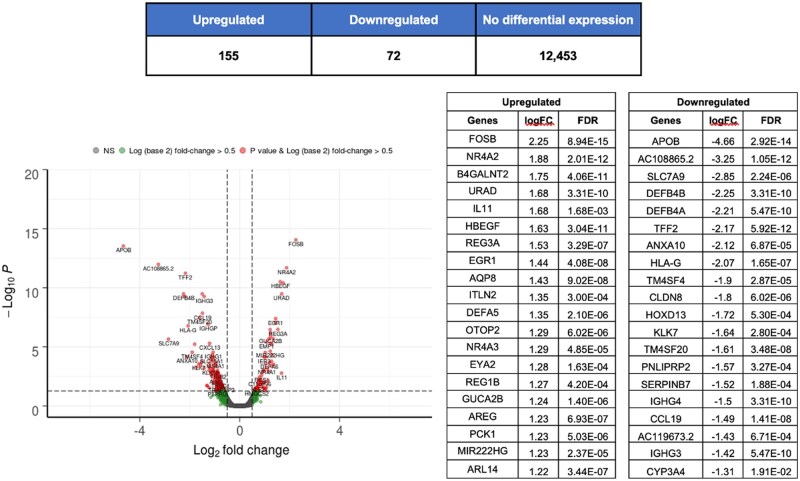
Fecal microbiota transplantation (FMT) response is marked by distinct mucosal transcriptomic profiles. Volcano plot showing differentially expressed genes in the colonic mucosa of responders between baseline and week 8. Red dots indicate significantly upregulated genes, and blue dots indicate significantly downregulated genes (FDR ≤ .05). The table lists the top 20 genes. Statistical analysis was performed using edgeR. See also Table S10.

Clinical response to FMT was marked by a profound transcriptomic shift in the colonic mucosa (*N* = 12), distinguishing responders from non-responders. Paired differential gene expression analysis from baseline to week 8 revealed that responders exhibited extensive changes, with 135 genes upregulated and 43 downregulated (Figure 9). Pathway analyses confirmed this was driven by an upregulation of processes related to cellular respiration, tissue remodeling, and SCFA metabolism. Concurrently, genes and pathways associated with proinflammatory immune activation, antimicrobial defense, and leukocyte trafficking were significantly downregulated (Table S10). In stark contrast, non-responders showed minimal transcriptomic modulation, with a modest upregulation of genes linked to proinflammatory mediators (Table S11). Direct week 8 comparisons further highlighted this divergence, with significant downregulation of genes related to antimicrobial and proinflammatory immune activation in responders compared to non-responders (Table S12). Collectively, these data demonstrate that clinical response to FMT is contingent on a fundamental reprogramming of the mucosal environment, shifting it from a persistent proinflammatory state to a metabolic and reparative one.

## 4. Discussion

This randomized trial provides critical insights into mechanistic underpinnings of FMT in UC. By comparing nasogastric and colonic routes of administration, the study highlights the superiority of colonic delivery, evidenced by higher efficacy and adherence rates with lower side-effect profile. Although the small cohort size limits the ability to draw conclusions about broader clinical efficacy, these findings are invaluable for informing future FMT study designs and advancing our understanding of the microbial and immunological effects that underpin its potential therapeutic effects.

The increased microbiome diversity observed in responders underscores the foundational role of microbial ecosystem restoration in UC. Responders showed significant enrichment of taxa from the class Clostridia, including *Ruminococcus*, *Oscillospirales*, and Christensenellaceae, some of which are associated with SCFA production and immune regulation. In particular, from MAGs we detected two strains increasing in responders that could be assigned to the butyrate-producing species *Mediterraneibacter butyricigenes.* From the 16S rRNA gene analysis we also found some related taxa that were positively correlated with gut-homing Tregs and negatively with proinflammatory Th17 cells. This could be tentative evidence for a direct mechanistic link between microbial composition and immune modulation, but these correlations would have to be confirmed by direct experiments.^25–28^ These findings align with emerging evidence that keystone microbial species act as critical modulators of host immunity, particularly through the production of SCFAs such as butyrate and propionate.^8^^,^^29–32^

The immune shifts observed in responders provide further evidence of the mechanistic impact of FMT. Significant increases in colonic mucosal Tregs, particularly gut-homing effector memory Tregs, and peripheral IL-10-producing CD4 T cells were observed, which are unlikely to be related to prior advanced therapy use (only three patients had a history of vedolizumab use). These changes were accompanied by reductions in Th17 and CD8 T cell populations, reflecting a transition to an anti-inflammatory immune state. Transcriptomic analysis corroborated these findings, revealing upregulation of pathways related to SCFA metabolism, cellular respiration, and tissue remodeling, coupled with the downregulation of genes involved in antimicrobial defense and proinflammatory immune activation. The contrast between responders and non-responders in these metrics highlights the importance of effective microbial engraftment and host–microbiota interactions in driving therapeutic outcomes. Lower fecal calprotectin levels in responders further emphasize the relationship between reduced intestinal inflammation and microbial and immunological changes. The observed negative correlation between calprotectin levels and alpha diversity, along with the robust increase in SCFA-producing taxa in responders, supports the hypothesis that microbial ecosystem restoration mediates inflammatory suppression in UC.

A major strength of this study is its detailed, multi-omic investigation of the mechanisms underlying FMT responses, integrated within a randomized design that provides practical insights into delivery route tolerability and engraftment dynamics. Our findings position FMT as a powerful model system to dissect host–microbiota interactions in UC and provide a clear rationale for developing next-generation, targeted microbial therapeutics. The specific enrichment of immunomodulatory Clostridia, known for their role in producing SCFAs such bas butyrate and educating the host immune system, suggests that defined microbial consortia could replicate the beneficial effects of FMT with improved safety, standardization, and scalability.^29^^,^^33^ Furthermore, the distinct immunological shifts observed in responders, such as the expansion of gut-homing Tregs, offer potential biomarkers for stratifying patients and monitoring responses in future clinical trials of microbiota-based interventions.

However, these findings must be interpreted in the context of the study’s limitations. The small cohort size, inherent to a pilot trial, restricts the generalizability of efficacy findings and limits the statistical power for subgroup analyses. The short duration of follow-up precludes the assessment of long-term impacts on the disease course, and the use of a limited number of donors might have obscured a potential association between donor characteristics and recipient response. Furthermore, while we present extensive immunological data, we acknowledge that these represent associations, and further studies are required to provide direct mechanistic proof of these immunological changes. Additionally, while this study compared delivery routes, the protocols also differed in the total volume, dose, and frequency of FMT administration (a single large bolus followed by enemas for the colonic route vs two pulsed courses of smaller infusions for the nasogastric route), and these factors are potential confounders that cannot be disentangled from the delivery route itself. Finally, participant dietary intake, a major potential confounder of the gut microbiome, was not monitored during the trial, and this study did not explore how baseline host factors, such as genetics or prior therapy exposure, may influence FMT outcomes, an area warranting further investigation.

In conclusion, while this study demonstrates the better acceptability of colonic FMT delivery, its greatest value lies in advancing our understanding of microbial and immunological effects in UC. These findings provide a roadmap for future research to refine FMT protocols, identify microbial and immunological targets, and develop novel microbial biotherapeutics that utilize the therapeutic potential of the gut microbiome. The insights gained here contribute to the growing body of evidence supporting biomarker-driven strategies for managing UC and highlight the promise of microbiota-based interventions in restoring gut homeostasis and modulating inflammation.

## Supplementary Material

jjag006_Supplementary_Data

## Data Availability

For further information and access to reagents or resources used in this study, please address the Lead Contact (t.h.iqbal@bham.ac.uk). All raw sequencing data and processed data files generated in this study have been deposited in the NCBI BioProject database under accession number PRJNA1283919. All accession numbers will be made publicly available as of the date of publication. MAGs and processed data files supporting the findings of this study are available within this deposition. This paper does not report original custom computer code or software. Publicly available software packages used for analysis are detailed in the Methods section. Any specific analysis scripts used for generating the reported results are available from the Lead Contact upon reasonable request.

## References

[1] Le Berre C , Honap S, Peyrin-Biroulet L. Ulcerative colitis. Lancet. 2023;402:571-584. 37573077 10.1016/S0140-6736(23)00966-2

[2] Kobayashi T , Siegmund B, Le Berre C, et al. Ulcerative colitis. Nat Rev Dis Primers.. 2020;6:74. 32913180 10.1038/s41572-020-0205-x

[3] Agrawal M , Allin KH, Petralia F, Colombel JF, Jess T. Multiomics to elucidate inflammatory bowel disease risk factors and pathways. Nat Rev Gastroenterol Hepatol. 2022;19:399-409. 35301463 10.1038/s41575-022-00593-y PMC9214275

[4] Shan Y , Lee M, Chang EB. The gut microbiome and inflammatory bowel diseases. Annu Rev Med. 2022;73:455-468. 34555295 10.1146/annurev-med-042320-021020 PMC10012812

[5] Zheng J , Sun Q, Zhang M, et al. Noninvasive, microbiome-based diagnosis of inflammatory bowel disease. Nat Med. 2024;30:3555-3567. 39367251 10.1038/s41591-024-03280-4 PMC11645270

[6] Danne C , Skerniskyte J, Marteyn B, Sokol H. Neutrophils: from IBD to the gut microbiota. Nat Rev Gastroenterol Hepatol. 2024;21:184-197. 38110547 10.1038/s41575-023-00871-3

[7] Lee M , Chang EB. Inflammatory bowel diseases (IBD) and the microbiome—searching the crime scene for clues. Gastroenterology. 2021;160:524-537. 33253681 10.1053/j.gastro.2020.09.056 PMC8098834

[8] Quraishi MN , Shaheen W, Oo YH, Iqbal TH. Immunological mechanisms underpinning faecal microbiota transplantation for the treatment of inflammatory bowel disease. Clin Exp Immunol. 2020;199:24-38. 31777058 10.1111/cei.13397 PMC6904658

[9] Gilliland A , Chan JJ, Wolfe TJD, Yang H, Vallance BA. Pathobionts in inflammatory bowel disease: origins, underlying mechanisms, and implications for clinical care. Gastroenterology. 2024;166:44-58. 37734419 10.1053/j.gastro.2023.09.019

[10] Palmela C , Chevarin C, Xu Z, et al. Adherent-invasive *Escherichia coli* in inflammatory bowel disease. Gut. 2018;67:574-587. 29141957 10.1136/gutjnl-2017-314903

[11] Britton GJ , Contijoch EJ, Mogno I, et al. Microbiotas from humans with inflammatory bowel disease alter the balance of gut Th17 and RORγt+ regulatory T cells and exacerbate colitis in mice. Immunity. 2019;50:212-224.e4. 30650377 10.1016/j.immuni.2018.12.015 PMC6512335

[12] Paramsothy S , Kamm MA, Kaakoush NO, et al. Multidonor intensive faecal microbiota transplantation for active ulcerative colitis: a randomised placebo-controlled trial. Lancet. 2017;389:1218-1228. 28214091 10.1016/S0140-6736(17)30182-4

[13] Moayyedi P , Surette MG, Kim PT, et al. Fecal microbiota transplantation induces remission in patients with active ulcerative colitis in a randomized controlled trial. Gastroenterology. 2015;149:102-109.e6. 25857665 10.1053/j.gastro.2015.04.001

[14] Rossen NG , Fuentes S, van der Spek MJ, et al. Findings from a randomized controlled trial of fecal transplantation for patients with ulcerative colitis. Gastroenterology. 2015;149:110-118.e4. 25836986 10.1053/j.gastro.2015.03.045

[15] Costello SP , Hughes PA, Waters O, et al. Effect of fecal microbiota transplantation on 8-week remission in patients with ulcerative colitis: a randomized clinical trial. JAMA. 2019;321:156-164. 30644982 10.1001/jama.2018.20046 PMC6439766

[16] Březina J , Bajer L, Wohl P. Fecal microbial transplantation versus mesalamine enema for treatment of active left-sided ulcerative colitis—results of a randomized controlled trial. J Clin Med. 2021;10:2753. 34206663 10.3390/jcm10132753 PMC8268406

[17] Kedia S , Virmani S, K Vuyyuru S, et al. Faecal microbiota transplantation with anti-inflammatory diet (FMT-AID) followed by anti-inflammatory diet al.ne is effective in inducing and maintaining remission over 1 year in mild to moderate ulcerative colitis: a randomised controlled trial. Gut. 2022;71:2401-2413. 35973787 10.1136/gutjnl-2022-327811

[18] Haifer C , Paramsothy S, Kaakoush NO, et al. Lyophilised oral faecal microbiota transplantation for ulcerative colitis (LOTUS): a randomised, double-blind, placebo-controlled trial. Lancet Gastroenterol Hepatol. 2022;7:141-151. 34863330 10.1016/S2468-1253(21)00400-3

[19] Tkach S , Dorofeyev A, Kuzenko I, et al. Efficacy and safety of fecal microbiota transplantation via colonoscopy as add-on therapy in patients with mild-to-moderate ulcerative colitis: a randomized clinical trial. Front Med (Lausanne). 2023;9:1049849. [cited 2025 Jan 12]; Available from: https://www.frontiersin.org/journals/medicine/articles/10.3389/fmed.2022.1049849/full 36714101 10.3389/fmed.2022.1049849 PMC9877446

[20] El Hage Chehade N , Ghoneim S, Shah S, et al. Efficacy of fecal microbiota transplantation in the treatment of active ulcerative colitis: a systematic review and meta-analysis of double-blind randomized controlled trials. Inflamm Bowel Dis. 2023;29:808-817. 35766805 10.1093/ibd/izac135

[21] Yalchin M , Segal JP, Mullish BH, et al. Gaps in knowledge and future directions for the use of faecal microbiota transplant in the treatment of inflammatory bowel disease. Ther Adv Gastroenterol. 2019;12:1756284819891038. 31803254 10.1177/1756284819891038 PMC6878609

[22] Rees NP , Shaheen W, Quince C, et al. Systematic review of donor and recipient predictive biomarkers of response to faecal microbiota transplantation in patients with ulcerative colitis. EBioMedicine. 2022;81:104088. 35660786 10.1016/j.ebiom.2022.104088 PMC9163485

[23] Quraishi MNN , Yalchin M, Blackwell C, et al. STOP-Colitis pilot trial protocol: a prospective, open-label, randomised pilot study to assess two possible routes of faecal microbiota transplant delivery in patients with ulcerative colitis. BMJ Open. 2019;9:e030659. 10.1136/bmjopen-2019-030659 PMC6858155 31719078

[24] Bingham SA , Welch AA, McTaggart A, et al. Nutritional methods in the European Prospective Investigation of Cancer in Norfolk. *Public Health Nutr*. 2001;4:847-858. 10.1079/phn2000102 11415493 11415493 10.1079/phn2000102

[25] Atarashi K , Tanoue T, Oshima K, et al. Treg induction by a rationally selected mixture of Clostridia strains from the human microbiota. Nature. 2013;500:232-236. 23842501 10.1038/nature12331

[26] Narushima S , Sugiura Y, Oshima K, et al. Characterization of the 17 strains of regulatory T cell-inducing human-derived Clostridia. Gut Microbes. 2014;5:333-339. 24642476 10.4161/gmic.28572 PMC4153770

[27] Atarashi K , Tanoue T, Shima T, et al. Induction of colonic regulatory T cells by indigenous *Clostridium* species. Science. 2011;331:337-341. 21205640 10.1126/science.1198469 PMC3969237

[28] Britton GJ , Contijoch EJ, Spindler MP, et al. Defined microbiota transplant restores Th17/RORγt+ regulatory T cell balance in mice colonized with inflammatory bowel disease microbiotas. Proc Natl Acad Sci U S A. 2020;117:21536-21545. 32817490 10.1073/pnas.1922189117 PMC7474624

[29] Zhao H , Zhou Y, Xu J, et al. Short-chain fatty acid-producing bacterial strains attenuate experimental ulcerative colitis by promoting M2 macrophage polarization via JAK/STAT3/FOXO3 axis inactivation. J Transl Med. 2024;22:369. 38637862 10.1186/s12967-024-05122-w PMC11025230

[30] Paramsothy S , Nielsen S, Kamm MA, et al. Specific bacteria and metabolites associated with response to fecal microbiota transplantation in patients with ulcerative colitis. Gastroenterology. 2019;156:1440-1454.e2. 30529583 10.1053/j.gastro.2018.12.001

[31] Deleu S , Machiels K, Raes J, Verbeke K, Vermeire S. Short chain fatty acids and its producing organisms: an overlooked therapy for IBD? EBioMedicine. 2021;66:103293. 33813134 10.1016/j.ebiom.2021.103293 PMC8047503

[32] Takeuchi T , Nakanishi Y, Ohno H. Microbial metabolites and gut immunology. Annu Rev Immunol. 2024;42:153-178. 38941602 10.1146/annurev-immunol-090222-102035

[33] Serebrinsky-Duek K , Barra M, Danino T, Garrido D. Engineered bacteria for short-chain-fatty-acid-repressed expression of biotherapeutic molecules. Microbiol Spectr. 2023;11:e00049-23. 36939337 10.1128/spectrum.00049-23 PMC10101121

