# Supplementary material for: Mechanistic insights into fecal microbiota transplantation for the treatment of ulcerative colitis: analysis of the STOP-Colitis trial
Source: J Crohns Colitis. 2026 Jan 23;20(3):jjag006. doi: 10.1093/ecco-jcc/jjag006 (PMC13012878; doi:10.1093/ecco-jcc/jjag006)
Supplement: jjag006_Supplementary_Data [file jjag006_supplementary_data.zip › Supplementary methods.docx]

**EXPERIMENTAL MODEL AND SUBJECT DETAILS**

**Human Subjects and Study Design**

The STOP-Colitis trial was a prospective, multicentre, open-label, randomized clinical trial designed to compare two different routes of faecal microbiota transplantation (FMT) for the treatment of active ulcerative colitis (UC). The study protocol is published in full (23). Participants were recruited from three hospitals in the United Kingdom: Queen Elizabeth Hospital in Birmingham, St Mark’s Hospital in London, and Glasgow Royal Infirmary.

Eligible participants were adults aged 16 to 70 years with active UC, defined by a Partial Mayo Score between 4 and 8 despite maintenance treatment with 5-aminosalicylates, with or without immunomodulators, or without any treatment (33). Key exclusion criteria included active infections, recent exposure (within protocol-specified timeframes) to advanced therapies (e.g., biologics, JAK inhibitors), antibiotics, probiotics, corticosteroids, or certain nutritional supplements. Additionally, pregnant or lactating participants were excluded. Baseline demographic and disease characteristics of the enrolled participants (N=30 randomized; 16 nasogastric, 14 colonic) are detailed in Table 1 of the main manuscript.

All participants provided written informed consent before enrolment and any study-related procedures. Ethical approval for the study was obtained from the East Midlands-Nottingham Research Ethics Committee (REC reference: 17/EM/0274). An independent trial oversight committee, including a Data Monitoring Committee and a Trial Steering Committee, provided monitoring and oversight of the trial. The trial was conducted in accordance with the Declaration of Helsinki and Good Clinical Practice guidelines.

**METHOD DETAILS**

**Trial Randomization and Interventions**

Participants were randomized in a 1:1 ratio using a computer-generated randomization sequence, with stratification based on baseline partial Mayo score (4–5 or 6–8) and smoking status (current smoker or non-smoker). Details of randomization were managed by the Birmingham Clinical Trials Unit.

**Donor Selection and Stool Processing:** FMT was produced at the University of Birmingham Microbiome Treatment Centre under a Good Manufacturing Practices (GMP) specials licence (MHRA MS 21761) following guidelines established by the British Society of Gastroenterology (34). Healthy volunteer donors underwent comprehensive screening, including questionnaires, clinical assessment, and extensive blood and stool testing for infectious agents. Each donor provided fresh stool samples across a 10-day collection period. Stool samples were processed within six hours of defecation under sterile, anaerobic conditions. The stool was homogenized with sterile 0.9% saline and 10% (v/v) glycerol (cryoprotectant), subsequently filtered through a [Specify Pore Size, e.g., 400µm] filter, aliquoted, and stored at −80°C until use. Each participant received FMT from a single, pre-assigned donor throughout their treatment.

**Bowel Preparation and FMT Administration:** Prior to the first FMT administration, all participants underwent bowel preparation with 2 litres of macrogol solution. Loperamide (4mg) was administered orally 30-60 minutes before and after each FMT infusion to promote retention.

- **Nasogastric Arm:** Participants received 50 mL of FMT (containing approximately 30 g of donor stool, based on wet weight) through a nasogastric tube, infused over [Specify duration, e.g., 15-30 minutes], on four consecutive days at baseline (Week 0). A second set of four daily nasogastric infusions was administered during Week 4. The total dose was approximately 240g of stool.
- **Colonic Arm:** The first FMT administration involved 250 mL of FMT (containing approximately 150 g of donor stool) delivered via colonoscopy to the caecum and ascending colon at Week 0. This was followed by weekly self-administered enemas of 100 mL FMT (containing approximately 30 g of stool) for the next seven weeks (Weeks 1-7). The total dose was approximately 360g of stool.

**Outcome Assessments and Sample Collection**

Participants were followed weekly up to Week 8 and then once more at Week 12. Partial Mayo scores, maintenance therapies, adverse events (AEs), and quality of life (QoL) measures (SF-36 and IBDQ questionnaires; (35,36) were assessed at each follow-up visit. Flexible sigmoidoscopies were performed at baseline (for eligibility confirmation if not done recently) and Week 8 to assess mucosal healing and calculate the full Mayo score (37). Faecal samples for microbiome and SCFA analysis were collected by participants at home and frozen immediately at -20°C before transport to the central laboratory on dry ice, where they were stored at -80°C. Samples were collected from recipients at baseline (pre-FMT), and at Weeks 2, 4, 6, 8, and 12 post-initial FMT. Donor stool samples (fresh and frozen aliquots from the 10-day collection period) were also processed and stored similarly. Colonic mucosal biopsies were obtained from the right colon, left colon, and rectum at baseline (if undergoing full colonoscopy for eligibility), and from the left colon and rectum at the Week 8 sigmoidoscopy. Biopsies for 16S rRNA gene sequencing and transcriptomics were placed in RNAlater TissueProtect Tubes (Qiagen) and stored at -80°C. Biopsies for immune cell analysis were processed fresh. Whole blood for PBMC isolation was collected into [Specify anticoagulant, e.g., EDTA or heparin tubes] at baseline and Week 8.

**Faecal DNA Extraction and 16S rRNA Gene Sequencing**

DNA was extracted from approximately 200 mg of faecal samples (donor and recipient time points) using the QIAamp PowerFaecal Pro DNA Kit (Qiagen) according to the manufacturer’s instructions. The V4 hypervariable region of the 16S rRNA gene was amplified using primers 515F (5’-GTGCCAGCMGCCGCGGTAA-3’) and 806R (5’-GGACTACHVGGGTWTCTAAT-3’) with Illumina overhang adapters and Golay barcodes for sample multiplexing. PCR amplification was performed using [Specify Polymerase, e.g., KAPA HiFi HotStart ReadyMix] with the following conditions: initial denaturation at 95°C for 3 min, followed by 30 cycles of 95°C for 30s, 55°C for 30s, and 72°C for 30s, and a final extension at 72°C for 5 min. PCR products were purified using Ampure XP beads (Beckman Coulter).

**16S rRNA Gene Sequencing Library Preparation and Bioinformatics**

Barcoded and purified amplicons were quantified, pooled in equimolar concentrations, and sequenced on an Illumina MiSeq platform using a 2 × 250 bp paired-end v2 kit. Each sequencing run included negative controls (extraction blanks and PCR no-template controls) and positive controls (ZymoBIOMICS Microbial Community Standard, Zymo Research). Raw 16S rRNA sequence data were processed using the DADA2 pipeline (v1.16) in R (v4.0.3). Forward and reverse reads were truncated at 240 bp and 200 bp respectively, based on quality profiles. Reads were filtered allowing a maximum expected error of 2. Non-templated nucleotides were removed by trimming the first 18 bases from forward reads and the first 2 bases from reverse reads. After error modeling, reads were denoised, paired-end reads merged, and an amplicon sequence variant (ASV) table was generated. Chimeric sequences were removed using the removeBimeraDenovo method. ASVs with lengths outside the range of 225–240 bp were excluded. ASVs were clustered into operational taxonomic units (OTUs) at 97% similarity using VSEARCH ([Specify Version]). Taxonomic classification of ASVs was performed to the genus level using the IDTAXA algorithm with the SILVA 16S reference database (release 138). Species-level annotations were added for sequences with 100% matches using the addSpecies function in DADA2.

**Faecal DNA Extraction and Metagenomic Sequencing**

For shotgun metagenomic sequencing, DNA was extracted from stool samples (donors and recipients at baseline, Weeks 2, 4, 8, and 12) as described for 16S rRNA sequencing. Metagenomic libraries were prepared using the NEBNext® Ultra™ II FS DNA Library Prep Kit (New England Biolabs) according to the manufacturer’s protocol, with single-indexing to enable paired-end sequencing. Sequencing was performed on the Illumina NovaSeq 6000 platform, generating 2 × 150 bp paired-end reads. Negative controls (extraction blanks) were included throughout DNA extraction and library preparation.

**Metagenomic Sequencing Bioinformatics**

Raw metagenomic reads underwent quality control: adapters were trimmed using Trimmomatic v0.39 (Bolger et al., 2014), and low-quality reads were filtered (Phred score < 20, minimum length 50 bp). Host (human) reads were removed by mapping to the human genome (GRCh38) using Bowtie2 ([Specify Version]). Taxonomic profiling was performed using MetaPhlAn4 with default parameters to quantify the relative abundance of microbial species (38). For de novo metagenome-assembled genome (MAG) construction, reads from each patient and their respective donor were co-assembled using MEGAHIT (v1.2.9) (39). Binning of contigs into MAGs was performed using CONCOCT (v1.1.0) (40) and MetaBat2 (v2.15) (41). MAG quality was assessed using CheckM (v1.1.3) (42) based on completeness (>50%) and contamination (<10%) using a panel of single-copy core genes. MAGs were dereplicated across patients at 99% average nucleotide identity (ANI) using dRep (v3.2.2) (43) to provide strain-level profiles, following normalization of coverage depths by total sequencing depth. MAGs were taxonomically annotated using GTDB-Tk (v1.7.0) against the GTDB database (Release R207) (44,45). Functional gene profiles were obtained by predicting open reading frames (ORFs) from assemblies using Prodigal (v2.6.3) (46). Predicted protein sequences were then aligned against the Kyoto Encyclopedia of Genes and Genomes (KEGG) functional gene database (Release [e.g., 109.0, Jan 2024]) using DIAMOND (v2.0.15) in sensitive mode (--sensitive) (47).

**Faecal Metabolomic Analysis (SCFA)**

Short-chain fatty acids (SCFAs: acetate, propionate, butyrate, valerate, caproate, heptanoate, caprylic acid, isobutyric acid, and isovaleric acid) were quantified from approximately 100-200 mg of faecal samples. Samples were acidified with [Specify Acid, e.g., 20% HCl or 1M H2SO4] and SCFAs were extracted by triple extraction with diethyl ether containing 2-ethylbutyric acid (74.0 mM) as an internal standard. The combined ether extracts were [Specify if derivatized, e.g., not derivatized or silylated with N-tert-Butyldimethylsilyl-N-methyltrifluoroacetamide (MTBSTFA)]. Analysis was performed by gas chromatography (Agilent 7890A) equipped with a flame ionization detector (FID) and a [Specify Column, e.g., Nukol™ fused silica capillary column, 15m x 0.53mm x 0.5µm film thickness, Supelco]. Helium was used as the carrier gas. The oven temperature program was [Specify program, e.g., initial 80°C for 1 min, ramp to 140°C at 10°C/min, hold for 5 min, then ramp to 200°C at 20°C/min, hold for 5 min]. SCFA concentrations were quantified against calibration curves generated from authentic external standards (acetic acid 185.8 mM, propionic acid 144.5 mM, butyric acid 114.2 mM, valeric acid 83.4 mM, caproic acid 52.6 mM, heptanoic acid 65.8 mM, caprylic acid 53.2 mM, isobutyric acid 97.3 mM, and isovaleric acid 87.0 mM). Samples from the same participant were analyzed in the same run, in duplicate. If the coefficient of variation (CV) exceeded 10%, a third replicate was performed. Concentrations were expressed as µmol/g of wet faecal matter.

**Colonic Biopsy Processing for Immune Cell Analysis (LPMCs)**

Lamina propria mononuclear cells (LPMCs) were isolated from fresh colonic mucosal biopsies (typically 4-6 biopsies per patient per timepoint) from 11 patients. Biopsies were incubated at 37°C for 1 hour with shaking (200 rpm) in gentleMACS C Tubes (Miltenyi Biotec) containing 5 mL of digestion mix: RPMI 1640 medium (Gibco) supplemented with 10% fetal bovine serum (FBS, Gibco), 1% Penicillin-Streptomycin (Gibco), 2mM L-glutamine (Gibco), 100 units/mL collagenase D (Roche), and 150 μg/mL DNase I (Sigma-Aldrich). Following incubation, samples were mechanically dissociated using the gentleMACS Dissociator (Miltenyi Biotec, program h_cord_01). The resulting cell suspension was passed through a 70 μm cell strainer (Falcon) and washed with PBS containing 2% FBS. LPMCs were isolated by density gradient centrifugation using 40%/80% Percoll (Sigma-Aldrich) gradient, resuspended in complete RPMI medium, and counted using a hemocytometer with Trypan Blue exclusion. A minimum yield of 1.5 million viable cells was targeted for flow cytometry.

**Peripheral Blood Mononuclear Cell (PBMC) Isolation**

Peripheral blood mononuclear cells (PBMCs) were isolated from 10-20 mL of whole blood (collected from 12 patients into EDTA tubes) within 4 hours of collection by density gradient centrifugation using Ficoll-Paque PLUS (GE Healthcare) or Histopaque-1077 (Sigma-Aldrich). Isolated PBMCs were washed twice with PBS and resuspended in complete RPMI medium for downstream analysis.

**Cell Stimulation and Flow Cytometry Staining**

Isolated LPMCs and PBMCs (typically 0.5-1 x 10^6 cells per panel) were aliquoted for CD4 phenotyping and intracellular cytokine staining panels, including fluorescence minus one (FMO) controls for key markers. For intracellular cytokine staining, cells were stimulated for 4 hours at 37°C, 5% CO2, using Cell Activation Cocktail with Brefeldin A (BioLegend, Cat# 423301), a pre-mixed solution containing phorbol 12-myristate-13-acetate (PMA; 50 ng/mL), ionomycin (1 µg/mL), and brefeldin A (10 µg/mL). After stimulation, cells were washed and stained for viability using Live-Dead Fixable Viability Stain (eBioscience/BD Biosciences, specific fluorochrome detailed with antibodies) for 20 min at 4°C in the dark. Surface marker staining was performed using a cocktail of fluorochrome-conjugated antibodies (detailed with antibodies) for 30 min at 4°C in the dark. Cells were then fixed and permeabilized using the BD FoxP3/Transcription Factor Staining Buffer Set (BD Biosciences, Cat# 562574/562725) according to the manufacturer's protocol. Intracellular and intranuclear staining (for cytokines and FoxP3) was performed using appropriate fluorochrome-conjugated antibodies for 30-45 min at 4°C in the dark. UltraComp eBeads™ Compensation Beads (eBioscience) were stained separately with each fluorochrome-conjugated antibody used in the panels to create single-stain compensation controls.

**Flow Cytometry Data Acquisition and Analysis**

Stained cells were acquired on a BD LSRFortessa™ Flow Cytometer (BD Biosciences) equipped with [Specify lasers, e.g., 4 lasers (blue, red, violet, yellow-green)] using BD FACSDiva™ software (v8.0 or higher). At least 10,000 events were recorded for compensation bead samples, and a target of 50,000-200,000 live, singlet lymphocyte events were recorded per LPMC/PBMC sample. Data analysis was performed using FlowJo™ software (v10.8, BD Life Sciences). Compensation was applied using the single-stain bead controls. Gating strategies involved initial gating on lymphocytes by forward scatter (FSC) and side scatter (SSC), followed by singlet gating (FSC-H vs FSC-A, SSC-H vs SSC-A), live cell gating (viability dye negative), and then specific immune cell populations based on marker expression.

**Colonic Biopsy Processing for Transcriptomics**

Colonic mucosal biopsies for transcriptomic analysis (from 11 patients, typically 2-3 biopsies pooled per patient per timepoint) were collected and immediately preserved in RNAlater TissueProtect Tubes (Qiagen) and stored at -20°C for up to 24 hours, then transferred to -80°C until processing. Total RNA was extracted within two weeks using the Qiagen AllPrep DNA/RNA Mini Kit (Qiagen) following mechanical lysis with the TissueLyser II (Qiagen; 2 min at 25 Hz with stainless steel beads). On-column DNase digestion was performed using the RNase-Free DNase Set (Qiagen) during RNA purification. RNA quantity was assessed using a Qubit Fluorometer (Thermo Fisher Scientific, Qubit RNA HS Assay Kit), and RNA integrity (RIN) was checked using the Agilent 2100 Bioanalyzer or 4200 TapeStation system (Agilent Technologies, RNA 6000 Nano/Pico Kit or High Sensitivity RNA ScreenTape). Only samples with RIN ≥ 7.0 were used for library preparation.

**RNA Sequencing and Transcriptomic Bioinformatics**

Ribosomal RNA (rRNA) was depleted from 100-500 ng of total RNA using the Ribo-Zero Gold Epidemiology rRNA Removal Kit (Illumina), which achieved ~95% removal of ribosomal RNA. Ribo-depleted RNA was cleaned via ethanol/glycogen precipitation using Pellet Paint® Co-Precipitant (MilliporeSigma). Strand-specific RNA sequencing libraries were prepared using the NEBNext Ultra II Directional RNA Library Prep Kit for Illumina (New England Biolabs, Cat# E7760) and sequenced on an Illumina NovaSeq 6000 platform, generating an average of 10.2 million 75 bp single-end reads per sample. Raw sequencing reads underwent quality control using FastQC (v0.11.9). Adapter sequences and low-quality bases were trimmed using AdapterRemoval v2 (v2.3.3) (48). Additional trimming, including sliding window trimming (window size 4, required quality 15) and removal of SmarTer Stranded Oligos (if applicable from library prep), was conducted using Trimmomatic v0.39 (49). Reads mapping to human ribosomal RNA sequences were removed by aligning to the Silva SSU/LSU ribosomal RNA database (v138.1, bacteria, archaea, and eukarya) using Bowtie2 (v2.4.5) with the “—very-sensitive-local” alignment option. After quality control and ribosomal RNA removal, an average of 9.9 million reads per sample remained. Cleaned reads were aligned to the human genome (GRCh38, Ensembl release 104 annotation) using STAR aligner (v2.5.3a) (50) with default parameters, except for allowing for more mismatches (--outFilterMismatchNmax 10) and reporting uniquely mapping reads. Gene-level read counts were quantified from BAM files using featureCounts (v2.0.3, part of the Subread package) (Liao et al., 2014), using Ensembl gene annotations (GRCh38.104.gtf). Only uniquely mapped reads assigned to exons were counted, and multi-mapping reads were excluded. Differential gene expression analysis was conducted using the edgeR package (v3.32.1) in R (v4.0.3) (51). Genes with fewer than 10 counts per million (CPM) in at least two libraries (the minimum group size) were excluded. Library sizes were normalized using the trimmed mean of M-values (TMM) method. Generalized linear models (GLMs) were applied to estimate dispersion using estimateDisp and glmQLFit. Differential expression between conditions or timepoints was assessed using the quasi-likelihood (QL) F-test (glmQLFTest). Genes were identified as significantly differentially expressed based on an FDR-adjusted p-value threshold of ≤0.05. Pathway analysis, including Gene Ontology (GO) biological processes and KEGG/Reactome pathways, was performed using Camera (competitive gene set test from the limma package v3.46.0) (52), using MSigDB gene sets. Pathways were considered significantly differentially expressed at an FDR-adjusted p-value threshold of ≤0.05.

**QUANTIFICATION AND STATISTICAL ANALYSIS**

Statistical analyses were performed using SAS version 9.4 (SAS Institute Inc., Cary, NC), Stata version 16 (StataCorp LLC, College Station, TX), R version 4.0.3 (R Core Team, Vienna, Austria) with specific packages as detailed herein, and GraphPad Prism version 8 (GraphPad Software, San Diego, CA). Baseline characteristics of study participants were described using means and standard deviations (SD) for normally distributed continuous variables, medians and interquartile ranges (IQR) for non-normally distributed continuous variables, and frequencies and percentages for categorical variables. Normality was assessed using Shapiro-Wilk tests and visual inspection of histograms. The primary outcome of the pilot trial (a composite qualitative assessment of feasibility and tolerability to select a route for a larger trial) was analyzed. Secondary clinical outcomes relevant to this mechanistic paper (clinical response, clinical remission, mucosal healing) were analyzed using a log-binomial regression model to calculate risk ratios (RR) and 95% confidence intervals (CI), adjusted for baseline Mayo score and smoking status. Adverse events were summarized descriptively as counts and percentages. Kaplan-Meier survival analysis was used to evaluate time to clinical response, with differences between treatment arms tested using the log-rank test or Cox proportional hazards regression where appropriate. All clinical efficacy analyses were performed on an intention-to-treat (ITT) basis, including all randomized participants who received at least one dose of FMT. For microbiome analyses, alpha diversity indices (observed 16S OTUs/ASVs, Shannon diversity from 16S data; observed species number, Shannon diversity from MetaPhlAn4 profiles) were calculated. 16S rRNA gene data were rarefied before alpha diversity calculation to correct for uneven sequencing depth. Changes in alpha diversity over time within groups (e.g., baseline vs. Week 8) were assessed using paired t-tests or Wilcoxon signed-rank tests. Differences in alpha diversity between independent groups (e.g., responders vs. non-responders at a specific timepoint) were assessed using two-sample t-tests or Mann-Whitney U tests. Beta diversity was assessed using Bray-Curtis dissimilarity matrices and visualized using Non-Metric Multidimensional Scaling (NMDS) plots. Permutational multivariate analysis of variance (PERMANOVA, using the adonis function in the R 'vegan' package, 999 permutations) was used to test for differences in overall microbial community composition between groups or time points, adjusting for relevant covariates where applicable. Differential abundance of taxa (OTUs/ASVs, MetaPhlAn4 species, MAGs) and KEGG orthologs/pathways between time points (paired analysis) or groups (unpaired analysis) was performed using appropriate tests (e.g., Wilcoxon signed-rank test for paired data, Mann-Whitney U test for independent groups, or DESeq2/edgeR for count data if appropriate) on relative abundances or normalized counts. P-values were adjusted for multiple comparisons using the Benjamini-Hochberg false discovery rate (FDR) procedure, with significance typically set at FDR < 0.1 or < 0.05 as specified in the text. Correlations between microbial taxa, pathways, SCFAs, and clinical/immunological markers (e.g., faecal calprotectin, immune cell frequencies) were assessed using Spearman's rank correlation coefficients (ρ or tau). For SCFA analysis, concentrations per gram of wet faecal matter were log-transformed to approximate normality before parametric testing. Comparisons between baseline and follow-up time points were made using paired t-tests. A repeated-measures mixed-effects model was applied to assess changes over all assessment time points (Weeks 2, 4, 6, 8, and 12), treating time as a categorical or continuous variable, and adjusting for baseline SCFA levels, smoking status, partial Mayo score, treatment allocation, and clinical response as fixed effects, with participant as a random effect. An unstructured covariance structure was used to account for correlations between repeated measurements on the same individual. Time-by-covariate interactions were assessed, and if significant (p ≤ 0.05), time-dependent effects were estimated. For immune cell data from flow cytometry, changes in cell frequencies from baseline to Week 8 were analyzed using paired t-tests or Wilcoxon signed-rank tests. Differences between responder and non-responder groups were assessed using two-sample t-tests or Mann-Whitney U tests. For transcriptomic data, differential gene expression was assessed using the quasi-likelihood (QL) F-test in edgeR, as described in the RNA sequencing bioinformatics section. Genes were considered significantly differentially expressed if the FDR-adjusted p-value was ≤0.05. Gene set enrichment analysis for GO terms and KEGG/Reactome pathways was performed using Camera, with pathways considered significantly differentially regulated at an FDR-adjusted p-value ≤0.05. Statistical significance for most analyses was set at a two-sided p-value < 0.05, or an FDR-adjusted p-value < 0.05 or < 0.1 as specified for multiple testing scenarios. Details of specific statistical tests used, exact values of n (representing number of participants or samples as appropriate), definitions of center (mean or median), and measures of dispersion and precision (SD, SEM, IQR, 95% CI) are provided in the main manuscript text, figure legends, and supplementary materials. The criteria for inclusion/exclusion of data or subjects are defined by the trial protocol and successful sample processing. No specific sample size estimation was performed for these mechanistic substudies as this was a pilot trial; analyses are exploratory.

**ADDITIONAL RESOURCES**

The detailed clinical trial protocol for the STOP-Colitis study has been published: Quraishi, M.N., Yalchin, M., Blackwell, C., Segal, J., Sharma, N., Hawkey, P., Ives, N., Magill, L., Quince, C., Gerasimidis, K., et al. (2019). STOP-Colitis pilot trial protocol: a prospective, open-label, randomised pilot study to assess two possible routes of faecal microbiota transplant delivery in patients with ulcerative colitis. BMJ Open 9, e030659.

Clinical trial registration: ISRCTN13636129; EudraCT Number: 2017-000805-34. The study received ethical approval from the East Midlands-Nottingham Research Ethics Committee (REC reference: 17/EM/0274).

**KEY RESOURCES TABLE**

The antibodies used in the study are detailed below

| Antibody | Fluorochrome | Supplier | Catalogue Number |
| --- | --- | --- | --- |
| Live-Dead | APC-CY7 | eBioscience | 65-0865-14 |
| Bcl2 | AF647 | Biolegend | 658705 |
| CCR5 | PE-Texas | Biolegend | 359126 |
| CCR6 | BV650 | Biolegend | 353425 |
| CCR7 | PeCF594 (PE-TR) | BD Biosciences | 562381 |
| CD127 | BV510 | Biolegend | 563036 |
| CD127 | PE-CY7 | Biolegend | 351319 |
| CD14 | APC-CY7 | Biolegend | 325620 |
| CD161 | BV711 | BD Biosciences | 563865 |
| CD19 | PE | Biolegend | 302208 |
| CD19 | APC-CY7 | Biolegend | 302217 |
| CD25 | BV785 | Biolegend | 302638 |
| CD25 | BV421 | BD Biosciences | 562443 |
| CD3 | FITC | Biolegend | 344804 |
| CD3 | BV605 | Biolegend | 317322 |
| CD3 | BV605 | Biolegend | 317322 |
| CD39 | BV711 | BD Biosciences | 563680 |
| CD4 | PerCP | Biolegend | 300528 |
| CD4 | BV650 | BD Biosciences | 563875 |
| CD4 | APC-CY7 | Biolegend | 300517 |
| CD45 | AF700 | Biolegend | 304023 |
| CD45 | BV510 | BD Biosciences | 563204 |
| CD45 | AF700 | Biolegend | 304023 |

| Antibody | Fluorochrome | Supplier | Catalogue Number |
| --- | --- | --- | --- |
| CD45RA | AF700 | Biolegend | 304119 |
| CD45RA | BV785 | Biolegend | 304139 |
| CD49d (a4 integrin) | PE | eBioscience | 12-0499-42 |
| CD8 | PE-CY7 | Biolegend | 344711 |
| CD8 | PECF594 | BD Biosciences | 562282 |
| CTLA4 | BV786 | BD Biosciences | 563931 |
| CXCR3 | BV421 | Biolegend | 353715 |
| FoxP3 | APC | eBioscience | 17-4776-41 |
| IFNy | BV650 | Biolegend | 502537 |
| IL-10 | PE | Biolegend | 501414 |
| IL13 | BV711 | BD Biosciences | 564288 |
| IL-17 | FITC | Biolegend | 512303 |
| IL-5 | BV421 | Biolegend | 504311 |
| Ki67 | PerCP | Biolegend | 652423 |
| Live-Dead | BV510 | BD Biosciences | 564406 |
| TNFa | APC | Biolegend | 502912 |

**RESOURCE AVAILABILITY**

**Lead Contact**

Further information and requests for resources and reagents should be directed to and will be fulfilled by the Lead Contact, Professor Tariq Iqbal (t.h.iqbal@bham.ac.uk).

**Materials Availability**

Faecal microbiota transplantation (FMT) material was produced at the University of Birmingham Microbiome Treatment Centre under a Good Manufacturing Practices specials licence (MHRA MS 21761) as described in the Method Details section. Specific aliquots used in this study are not available for redistribution due to regulatory and ethical constraints associated with human-derived materials. Other materials used are commercially available and would be detailed in a Key Resources Table.

**Data and Code Availability**

**Resource availability**

For further information and access to reagents or resources used in this study, please address the Lead Contact (t.h.iqbal@bham.ac.uk).

Data and Code Availability

Raw 16S rRNA gene sequencing data, shotgun metagenomic sequencing data, and colonic mucosal transcriptomic data generated during this study have been deposited in the NCBI BioProject database under accession number PRJNA1283919. All accession numbers will be made publicly available as of the date of publication. Metagenome Assembled Genomes (MAGs) and processed data files supporting the findings of this study are available within these depositions or can be obtained from the Lead Contact upon reasonable request. This paper does not report original custom computer code or software. Publicly available software packages used for analysis are detailed in the Method Details sections and would be listed in a Key Resources Table. Any specific analysis scripts used for generating the reported results are available from the Lead Contact upon reasonable request. Any additional information required to reanalyse the data reported in this paper is available from the Lead Contact upon request.
