# Supplementary material for: Mechanistic insights into fecal microbiota transplantation for the treatment of ulcerative colitis: analysis of the STOP-Colitis trial
Source: J Crohns Colitis. 2026 Jan 23;20(3):jjag006. doi: 10.1093/ecco-jcc/jjag006 (PMC13012878; doi:10.1093/ecco-jcc/jjag006)
Supplement: jjag006_Supplementary_Data [file jjag006_supplementary_data.zip › Supplementary Tables.docx]

**Supplementary table 1**

**3% 16S rRNA gene OTUs in donor sample associating with response.** The results are from t tests on log-transformed relative abundances. The p-values (p) were adjusted with Benjamini-Hochberg for multiple comparisons (pa). Natural log means in the responder and non-responder donor group samples are also shown (meanR and meanNR respectively).


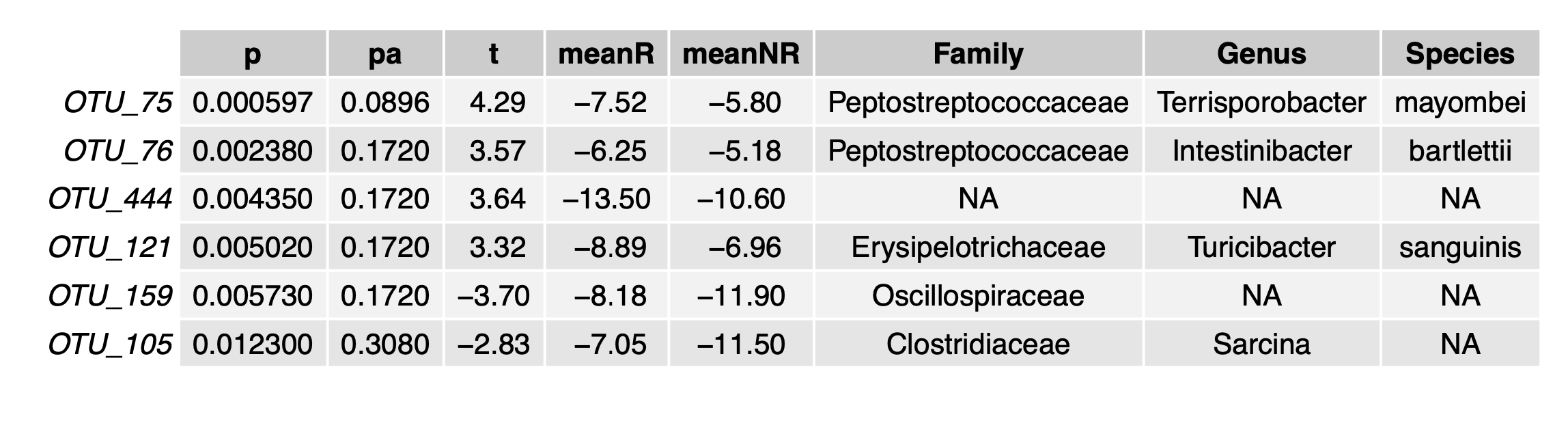


**Supplementary table 2**

**Changes in metagenome species number over time in the patient groups.** The results are for paired t-tests for changes in patient fecal metagenome species number from MetaPhlAn4 between day 1 and the day given in the corresponding row of the table split across three groups, all patients, responders and non-responders.


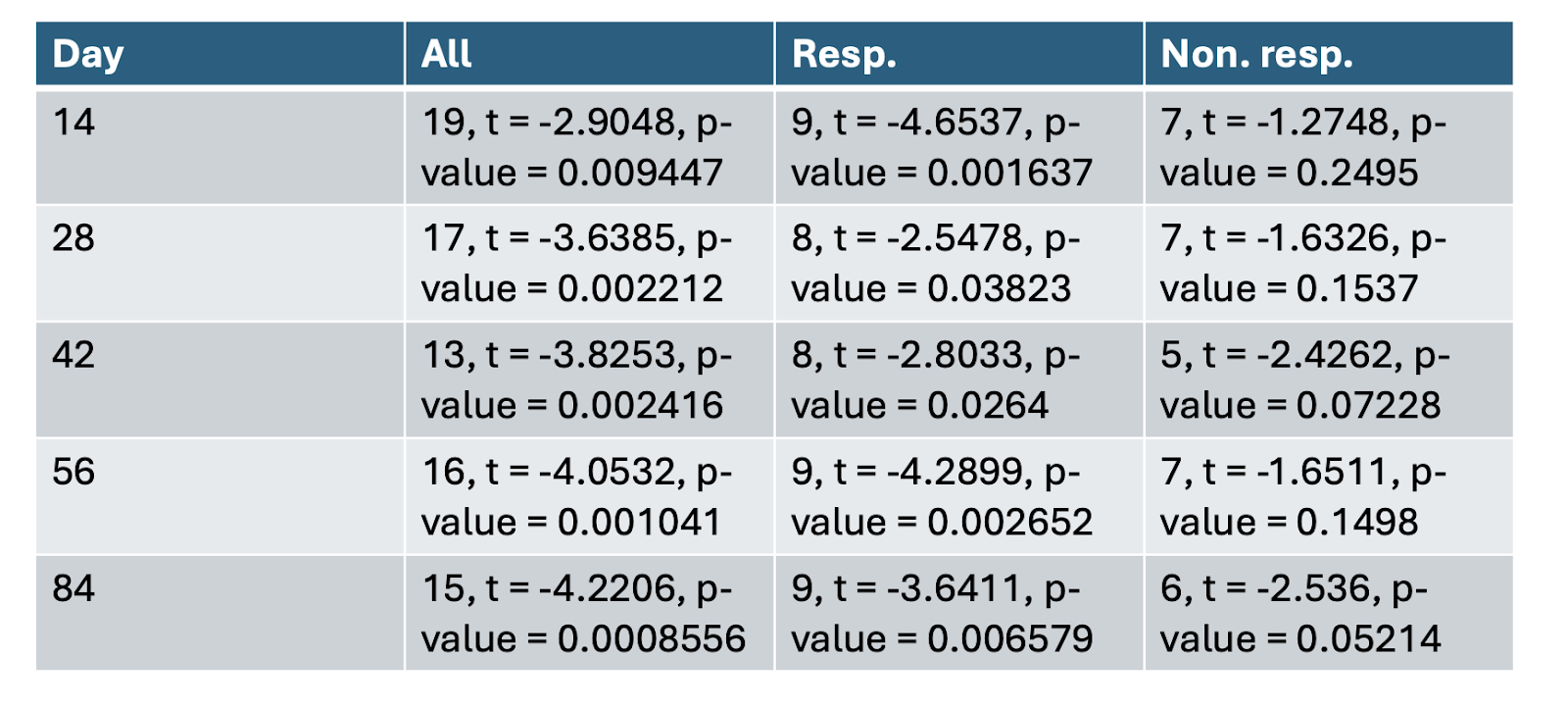


**Supplementary table 3**

**3% OTUs that changed significantly in abundance from day 1 to day 56 in all patients.** The results are for paired t-tests for changes in patient log 3% OTU abundance together with the fraction of patients for which this OTU had non-zero abundance at the two time points (f1) and (f56) for day 1 and day 56 respectively. We give the test statistic (chi) and p-values (p) and adjusted (pa) values as the Benjamini-Hochberg FDR.


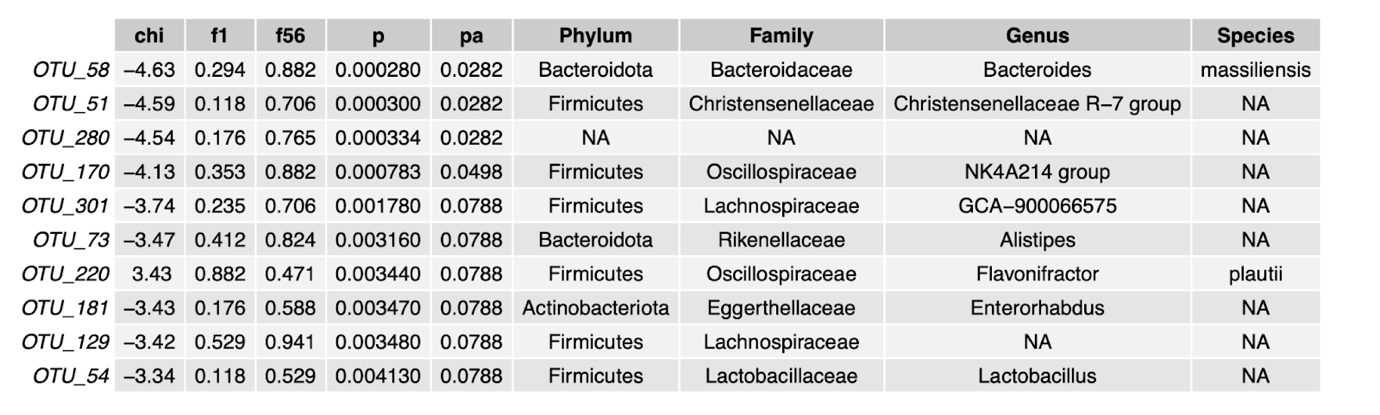


**Supplementary table 4**

**Top 10 MetaPhlAn4 species that changed significantly in abundance from day 1 to day 56 in all patients (N = 16).** The results are for paired t-tests for changes in patient log 3% OTU abundance together with the fraction of patients for which this OTU had non-zero abundance at the two time points (f1) and (f56) for day 1 and day 56 respectively. We give the test statistic (chi) and p-values (p) and adjusted (pa) values as the Benjamini-Hochberg FDR.


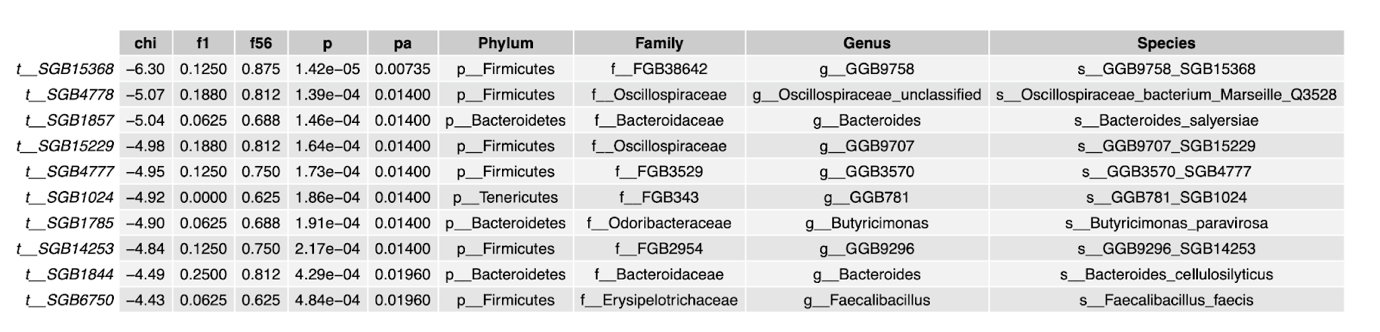


**Supplementary table 5**

**Top 10 MetaPhlAn4 species that changed significantly in abundance from day 1 to day 56 in the Responders only (N = 9).** The results are for paired t-tests for changes in patient log species abundance together with the fraction of patients for which this species had non-zero abundance at the two time points (f1) and (f56) for day 1 and day 56 respectively. We give the test statistic (chi) and p-values (p) and adjusted (pa) values as the Benjamini-Hochberg FDR.


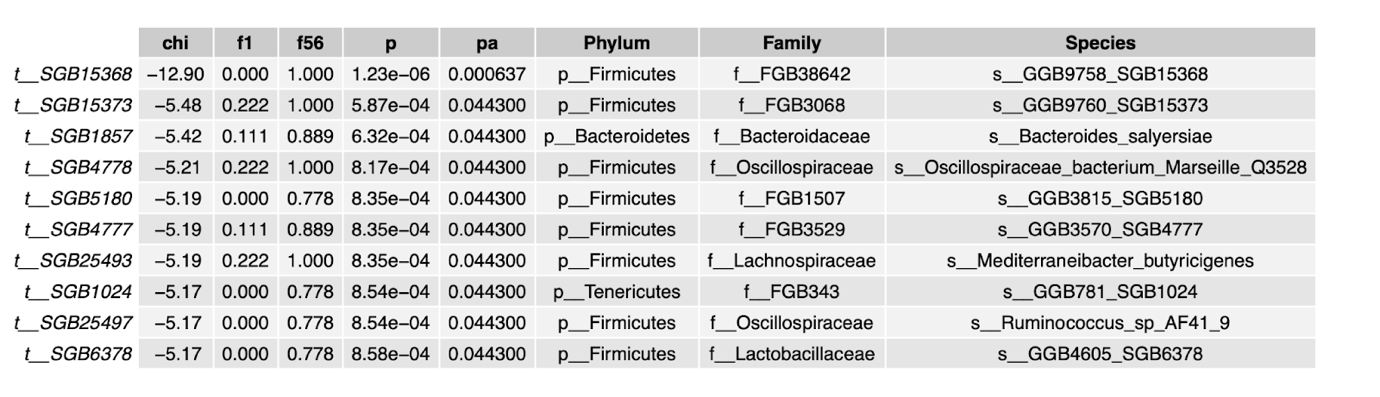


**Supplementary table 6**

**Top 10 metagenome assembled genomes dereplicated to 99% nucleotide identity (dMAGs) that changed significantly in abundance from day 1 to day 56 in the Responders only (N = 9).** The results are for paired t-tests for changes in patient log dMAG abundance together with the fraction of patients for which this dMAG had non-zero abundance at the two time points (f1) and (f56) for day 1 and day 56 respectively. We give the test statistic (chi) and p-values (p) and adjusted (pa) values as the Benjamini-Hochberg FDR.


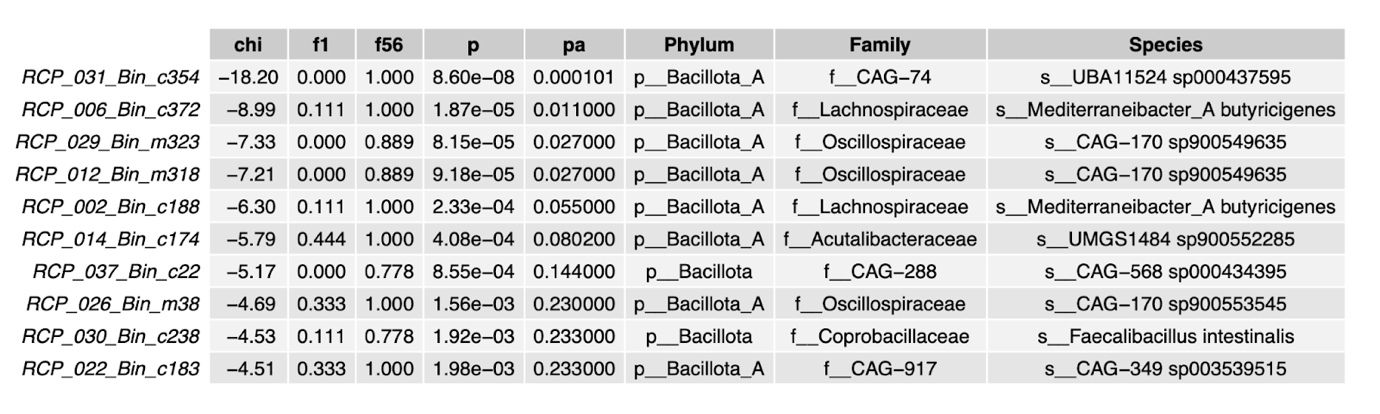


**Supplementary table 7**

**3% 16S rRNA gene OTUs in recipient samples associating with faecal calprotectin.** The results are from Pearson’s correlation. The p-values (p) were adjusted with Benjamini-Hochberg for multiple comparisons (pa).


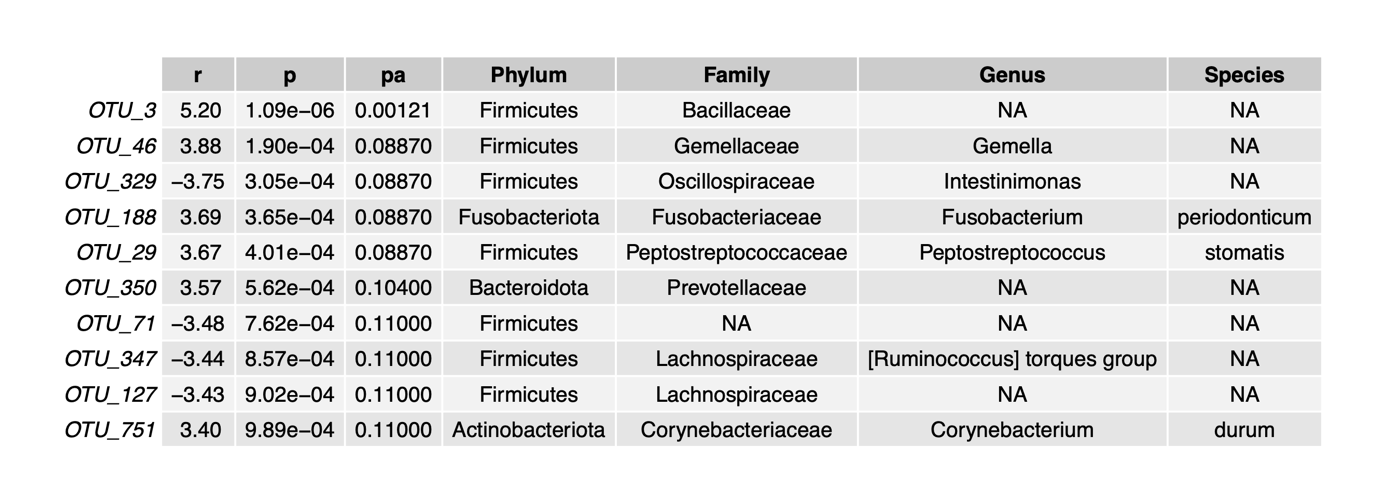


**Supplementary table 8**

**3% 16S rRNA gene OTUs in recipient samples associating with Gut-homing Treg populations.** For each 3% OTU that was found in at least four of eleven recipients we correlated change in Gut-homing Treg abundance with change in relative abundance of the OTU between days 1 and 56. We show the test statistic (chi) and p-values from a glm together with the Benjamini-Hochberg adjusted p-values and the OTU phyla, family, genera and species if available.


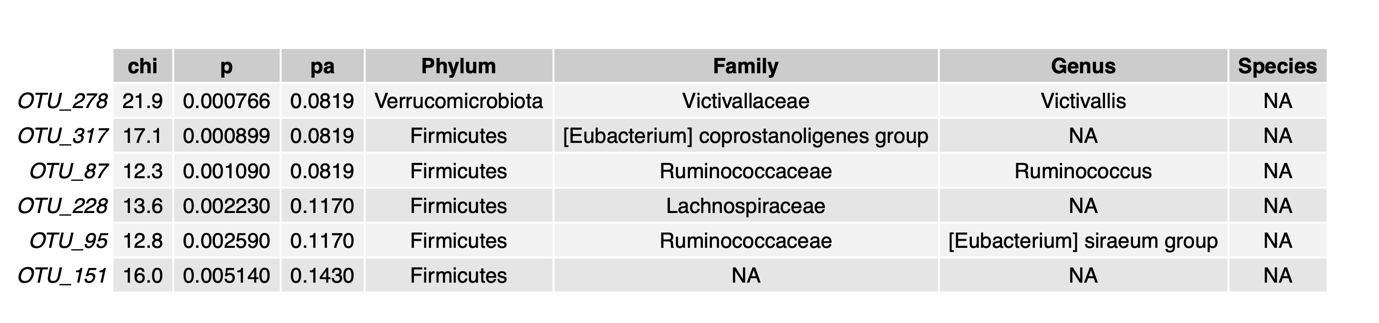


**Supplementary table 9**

**3% 16S rRNA gene mucosal OTUs changing with treatment in recipient samples.** We determined OTUs that changed as a result of the FMT treatment across 18 individuals whilst account for subject level differences using the glm function of R. The p-values (p) were adjusted for multiple comparisons using Benjami-Hochberg (pa).


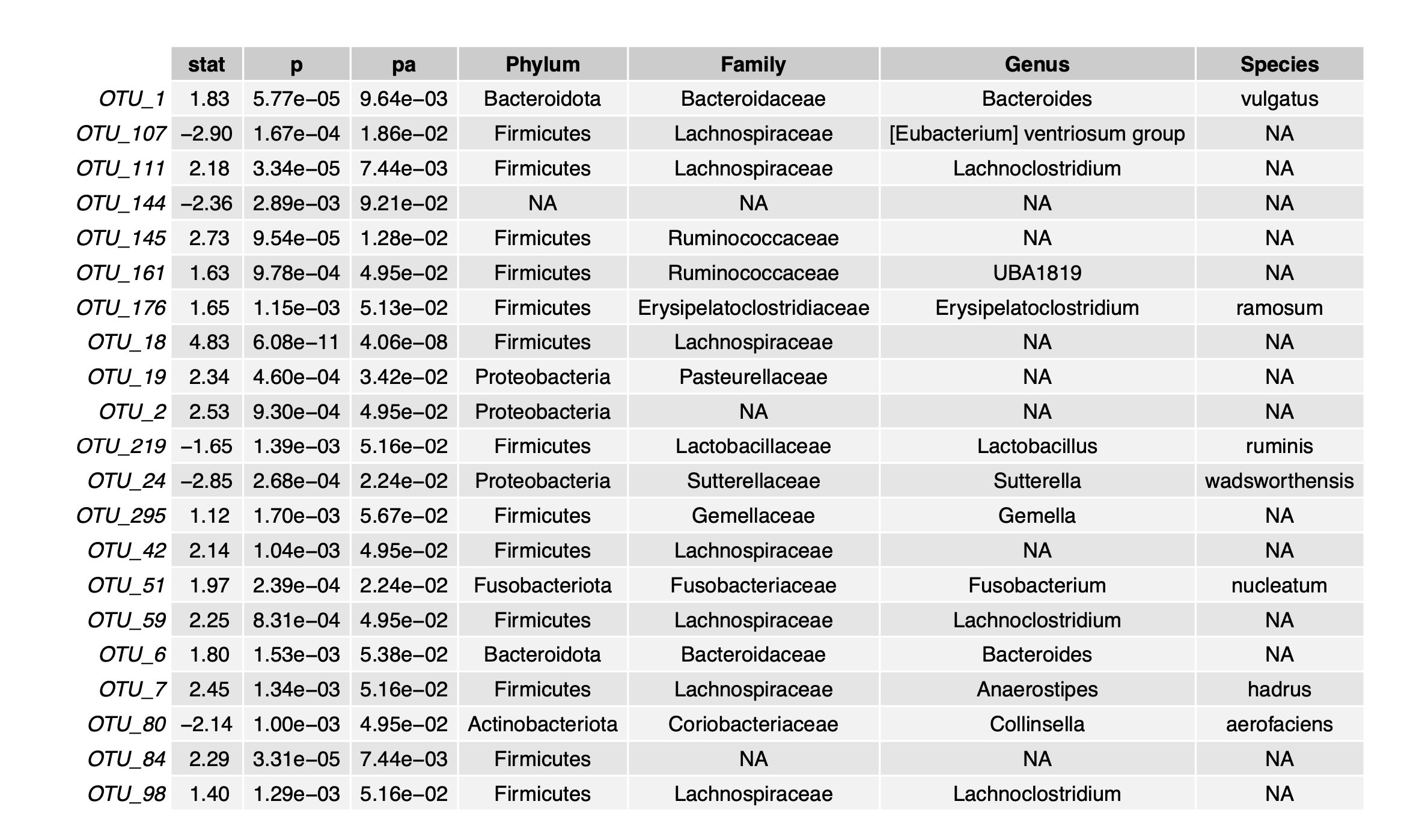


**Supplementary table 10**

**Differentially expressed genes in the colonic mucosa of responders between Baseline and Week 8.**

| **Genes** | **logFC** | **PValue** | **FDR** |
| --- | --- | --- | --- |
| APOB | -5.6485466 | 9.75E-23 | 5.86E-19 |
| AC108865.2 | -3.7914722 | 1.11E-11 | 1.49E-08 |
| SLC7A9 | -2.8242296 | 2.11E-08 | 8.75E-06 |
| KLK7 | -2.81255 | 7.02E-10 | 6.49E-07 |
| TM4SF4 | -2.4479312 | 5.84E-09 | 3.05E-06 |
| TM4SF20 | -2.4269149 | 1.02E-15 | 2.03E-12 |
| ANXA10 | -2.4120061 | 2.72E-06 | 0.00059426 |
| TFF2 | -2.2261001 | 2.19E-12 | 3.29E-09 |
| DEFB4B | -2.1811266 | 5.84E-09 | 3.05E-06 |
| DEFB4A | -2.1116227 | 1.43E-08 | 6.89E-06 |
| TCN1 | -2.0510726 | 1.52E-08 | 7.04E-06 |
| S100A8 | -1.980592 | 5.56E-11 | 5.56E-08 |
| HLA-G | -1.9426612 | 1.15E-06 | 0.00027537 |
| CYP3A4 | -1.7944113 | 1.20E-06 | 0.00028378 |
| GLDN | -1.7292575 | 1.96E-09 | 1.31E-06 |
| AC022034.1 | -1.7201473 | 5.51E-09 | 3.05E-06 |
| S100A9 | -1.6956678 | 2.37E-09 | 1.50E-06 |
| KLK10 | -1.6845769 | 1.76E-07 | 5.87E-05 |
| IGHG4 | -1.6437682 | 5.43E-11 | 5.56E-08 |
| SAA1 | -1.6289624 | 2.34E-07 | 7.23E-05 |
| GNLY | -1.5254329 | 3.88E-07 | 0.00011088 |
| MIA | -1.506299 | 3.06E-06 | 0.00064112 |
| SERPINB7 | -1.4969331 | 0.00015712 | 0.01492531 |
| FABP6 | -1.4700693 | 0.00015123 | 0.0146653 |
| IGHG3 | -1.4657977 | 7.89E-10 | 6.77E-07 |
| MMP7 | -1.4512947 | 1.47E-05 | 0.00215631 |
| KRT17 | -1.4448712 | 0.00021237 | 0.0187637 |
| TNIP3 | -1.4434156 | 5.76E-07 | 0.00016099 |
| CCL23 | -1.4401266 | 1.14E-06 | 0.00027537 |
| CLDN8 | -1.4377595 | 2.00E-05 | 0.00278458 |
| LAIR2 | -1.4348355 | 0.00071678 | 0.05066363 |
| CCL19 | -1.4326058 | 1.46E-07 | 5.02E-05 |
| CHI3L1 | -1.412392 | 7.79E-06 | 0.00129964 |
| IGHG1 | -1.4110441 | 1.80E-09 | 1.27E-06 |
| GZMA | -1.3849681 | 1.73E-08 | 7.70E-06 |
| CSF3R | -1.3767197 | 1.14E-05 | 0.0017142 |
| SLC6A14 | -1.3671932 | 2.37E-07 | 7.23E-05 |
| SAA2 | -1.361041 | 1.62E-05 | 0.00231282 |
| SLC15A1 | -1.3555485 | 3.40E-08 | 1.32E-05 |
| FCGR3B | -1.3522245 | 5.04E-05 | 0.00624838 |
| PI3 | -1.3427201 | 4.62E-08 | 1.74E-05 |
| VNN1 | -1.3302278 | 2.19E-06 | 0.00049582 |
| DUOXA2 | -1.3280924 | 6.56E-06 | 0.00117667 |
| CXCR2 | -1.32773 | 0.00028373 | 0.02335177 |
| CCL20 | -1.3203595 | 2.41E-07 | 7.23E-05 |
| ANO5 | -1.3155103 | 9.55E-05 | 0.01052106 |
| DUOX2 | -1.3025524 | 6.05E-07 | 0.00016532 |
| CCL5 | -1.2354951 | 6.29E-08 | 2.29E-05 |
| MYRF | -1.2351651 | 4.74E-06 | 0.00087621 |
| RASGRP1 | -1.2319486 | 2.79E-06 | 0.0005977 |
| SULT1C2 | -1.223424 | 4.06E-05 | 0.00524689 |
| CD247 | -1.2196006 | 3.09E-06 | 0.00064112 |
| TMPRSS3 | -1.2187797 | 0.00015134 | 0.0146653 |
| XPNPEP2 | -1.2138704 | 0.00019889 | 0.01783511 |
| IGHG2 | -1.2095459 | 2.24E-07 | 7.23E-05 |
| PPY | -1.2029078 | 0.00062914 | 0.04526829 |
| NOS2 | -1.2023266 | 3.47E-05 | 0.00458749 |
| CXCL9 | -1.1845831 | 1.04E-05 | 0.00160689 |
| UBD | -1.1802379 | 8.06E-06 | 0.00132643 |
| IGHGP | -1.1764423 | 7.21E-07 | 0.00019164 |
| GPR82 | -1.1589783 | 6.22E-05 | 0.00754915 |
| C8G | -1.1563128 | 0.00051458 | 0.03840481 |
| CXCL1 | -1.156244 | 1.64E-05 | 0.00231282 |
| SLFN12L | -1.1534289 | 0.00031123 | 0.02509864 |
| CXCL13 | -1.1513515 | 4.87E-06 | 0.00088655 |
| MMP3 | -1.1492521 | 0.00025987 | 0.0218367 |
| LST1 | -1.1412685 | 4.22E-05 | 0.00540062 |
| GABBR1 | -1.1278569 | 7.28E-06 | 0.00128581 |
| MTND4P24 | -1.1228618 | 0.00079213 | 0.05438972 |
| PTPN7 | -1.1136332 | 1.06E-05 | 0.00161507 |
| MTCL1 | -1.1121503 | 0.0001671 | 0.01556475 |
| C3 | -1.1113105 | 3.64E-06 | 0.00072985 |
| PRSS22 | -1.1073822 | 8.80E-06 | 0.00142858 |
| LEAP2 | -1.1054434 | 0.00040334 | 0.03126817 |
| SLC6A20 | -1.1022816 | 2.36E-05 | 0.00318126 |
| TNFRSF6B | -1.0940304 | 2.70E-05 | 0.00361061 |
| CD3D | -1.0913466 | 9.04E-06 | 0.00144886 |
| IGHV3-30 | -1.0894802 | 0.00026677 | 0.02210668 |
| CXCL11 | -1.088526 | 0.00049994 | 0.03754584 |
| G0S2 | -1.0850218 | 0.00021154 | 0.0187637 |
| AATK | -1.0698285 | 7.29E-05 | 0.00850356 |
| SNX10 | -1.0691101 | 1.04E-05 | 0.00160689 |
| MMP9 | -1.065931 | 7.43E-06 | 0.00129461 |
| APOL1 | -1.0624794 | 2.28E-05 | 0.0031187 |
| AIM2 | -1.0603284 | 0.00014119 | 0.01417723 |
| LCN2 | -1.0583676 | 0.00013195 | 0.01346467 |
| TNFSF14 | -1.057828 | 0.00024118 | 0.02040861 |
| LRRK2 | -1.0522059 | 0.0001648 | 0.01547057 |
| PLAAT2 | -1.052003 | 9.63E-05 | 0.01052106 |
| FCGR3A | -1.0486267 | 0.0002665 | 0.02210668 |
| CD3G | -1.03697 | 9.50E-06 | 0.00150196 |
| RTEL1-TNFRSF6B | -1.0312999 | 4.71E-05 | 0.00589072 |
| AC092723.1 | -1.0255511 | 0.00080239 | 0.05451638 |
| TRIB2 | -1.0226979 | 2.02E-05 | 0.00278458 |
| CD7 | -1.021915 | 8.40E-05 | 0.00942852 |
| PYHIN1 | -1.0114064 | 0.00034581 | 0.02751804 |
| MIAT | -0.999521 | 0.00010841 | 0.01163052 |
| TFF1 | -0.9869648 | 3.49E-06 | 0.00071006 |
| IRF4 | -0.9853086 | 0.00014814 | 0.01459085 |
| CREB3L3 | -0.9762641 | 0.00044524 | 0.03429505 |
| CTSE | -0.9708459 | 7.64E-06 | 0.00129753 |
| ABCC13 | -0.9705822 | 7.66E-05 | 0.00885585 |
| LOXL1 | -0.9672834 | 0.000229 | 0.01979615 |
| CD6 | -0.9597553 | 0.00039758 | 0.03102124 |
| PCED1B-AS1 | -0.9593503 | 0.00013165 | 0.01346467 |
| PLAAT4 | -0.9592271 | 4.62E-05 | 0.00584959 |
| ITGAX | -0.9504581 | 0.00057166 | 0.04163044 |
| FCRL5 | -0.939265 | 0.00014158 | 0.01417723 |
| ANO6 | -0.9358712 | 3.88E-05 | 0.00507264 |
| SELL | -0.9282968 | 0.00011476 | 0.01220328 |
| MS4A1 | -0.9246929 | 6.88E-05 | 0.00818471 |
| ACAP1 | -0.9196602 | 0.00035287 | 0.0278954 |
| TGM2 | -0.9178828 | 6.96E-05 | 0.00819893 |
| IFIT3 | -0.9166344 | 0.00014694 | 0.01459085 |
| PRAC1 | -0.9155815 | 6.36E-05 | 0.00764607 |
| ITPR1 | -0.8968972 | 0.00015775 | 0.01492531 |
| IKZF3 | -0.8884231 | 8.87E-05 | 0.00986422 |
| NLRP1 | -0.880551 | 0.00073333 | 0.05093479 |
| APOL2 | -0.8761938 | 0.00024016 | 0.02040861 |
| CCL21 | -0.8726503 | 0.00036235 | 0.02845758 |
| HLA-DOA | -0.866631 | 0.00046868 | 0.03541899 |
| HOXB13 | -0.8483052 | 0.00018774 | 0.01708971 |
| GMFG | -0.8454527 | 0.00029873 | 0.0244186 |
| SI | -0.8397166 | 0.00022259 | 0.01952279 |
| NCF2 | -0.837144 | 0.00045486 | 0.03481278 |
| ADM | -0.8316102 | 0.00017144 | 0.01572556 |
| TRBC1 | -0.8302459 | 0.00031074 | 0.02509864 |
| GREM1 | -0.8136631 | 0.00053002 | 0.03931273 |
| IL7R | -0.8110516 | 0.00015383 | 0.01478733 |
| IFI6 | -0.7978091 | 0.00024046 | 0.02040861 |
| IGLC3 | -0.7628418 | 0.0005623 | 0.04119903 |
| SPOCK2 | -0.7609961 | 0.00074476 | 0.05143114 |
| IGLC2 | -0.7499151 | 0.00069153 | 0.04916784 |
| PPP1R18 | -0.7399324 | 0.00067105 | 0.04799595 |
| SLC40A1 | -0.7174057 | 0.00080305 | 0.05451638 |
| ZNF331 | 0.78683681 | 0.00073081 | 0.05093479 |
| EMP1 | 0.79527318 | 0.00013223 | 0.01346467 |
| IRS2 | 0.80623184 | 0.00017051 | 0.01572556 |
| AVPI1 | 0.82927701 | 0.00012178 | 0.01272442 |
| AC129492.1 | 0.83774767 | 0.0004659 | 0.03541899 |
| GUCA2A | 0.87898871 | 5.50E-05 | 0.00674479 |
| TNNC2 | 0.88919736 | 0.00054597 | 0.04024739 |
| FAM81A | 0.89431337 | 0.00059127 | 0.04279972 |
| CDHR1 | 0.92902378 | 8.20E-05 | 0.00929667 |
| PTGS2 | 0.93038038 | 0.00022745 | 0.01979615 |
| GCNT2 | 0.95012637 | 0.00012156 | 0.01272442 |
| OTOP2 | 0.9551859 | 0.000108 | 0.01163052 |
| PDE6A | 0.9577418 | 0.0003346 | 0.02680393 |
| GUCA2B | 1.00001367 | 7.67E-06 | 0.00129753 |
| TCIM | 1.04865485 | 1.59E-05 | 0.00230831 |
| C11orf96 | 1.0737639 | 3.76E-06 | 0.00074068 |
| ARL14 | 1.07960245 | 2.47E-06 | 0.0005489 |
| TPPP3 | 1.10966305 | 7.84E-05 | 0.00897064 |
| RGS1 | 1.13500396 | 4.55E-06 | 0.00085476 |
| AQP8 | 1.13995268 | 1.45E-06 | 0.00033451 |
| IER3 | 1.15830409 | 2.61E-07 | 7.66E-05 |
| XIST | 1.21714353 | 0.00072934 | 0.05093479 |
| AREG | 1.31352232 | 6.83E-08 | 2.41E-05 |
| PCK1 | 1.31640822 | 3.00E-08 | 1.20E-05 |
| ADAMTS1 | 1.31937699 | 1.11E-06 | 0.00027537 |
| MOCS1 | 1.3305311 | 1.23E-05 | 0.00181753 |
| ITLN2 | 1.33571031 | 0.0001917 | 0.01731964 |
| FOS | 1.42806318 | 3.52E-09 | 2.11E-06 |
| ACKR3 | 1.43231917 | 4.04E-06 | 0.00077088 |
| URAD | 1.45400572 | 2.09E-08 | 8.75E-06 |
| ATF3 | 1.48072211 | 9.51E-10 | 7.62E-07 |
| EGR3 | 1.48416871 | 1.10E-06 | 0.00027537 |
| NR4A3 | 1.52475324 | 7.34E-07 | 0.00019164 |
| ASPG | 1.57130716 | 4.02E-06 | 0.00077088 |
| MIR222HG | 1.58305301 | 7.27E-09 | 3.64E-06 |
| NR4A1 | 1.89879489 | 2.37E-11 | 2.84E-08 |
| B4GALNT2 | 1.92060886 | 6.51E-14 | 1.12E-10 |
| HBEGF | 2.10437806 | 5.79E-16 | 1.39E-12 |
| LINC00923 | 2.24576397 | 1.04E-09 | 7.81E-07 |
| EGR1 | 2.34757167 | 5.62E-18 | 1.69E-14 |
| NR4A2 | 2.55896118 | 8.00E-21 | 3.20E-17 |
| FOSB | 3.27515072 | 1.68E-28 | 2.01E-24 |

**Supplementary table 11**

**Differentially expressed genes in the colonic mucosa of non-responders between Baseline and Week 8.**

| **Genes** | **logFC** | **PValue** | **FDR** |
| --- | --- | --- | --- |
| HCAR3 | 3.328676 | 9.34E-15 | 1.13E-10 |
| DEFA5 | 3.270669 | 1.26E-13 | 7.61E-10 |
| HCAR2 | 2.935347 | 1.87E-11 | 7.50E-08 |
| REG3A | 2.721027 | 3.27E-11 | 9.87E-08 |
| IL11 | 2.614029 | 3.98E-06 | 0.001819 |
| OASL | 2.574034 | 1.62E-09 | 3.91E-06 |
| TMIGD1 | 2.549082 | 8.18E-08 | 9.86E-05 |
| USP2 | 2.407224 | 1.83E-08 | 2.61E-05 |
| SLC30A10 | 2.378331 | 9.99E-07 | 0.000927 |
| CLDN23 | 2.371934 | 1.37E-06 | 0.00101 |
| AQP8 | 2.362112 | 2.70E-06 | 0.001546 |
| REG1A | 2.306791 | 1.09E-07 | 0.000119 |
| DEFA6 | 2.286257 | 1.94E-08 | 2.61E-05 |
| OTOP2 | 2.285207 | 3.15E-06 | 0.001582 |
| ABCG2 | 2.189427 | 9.33E-07 | 0.000927 |
| URAD | 2.181596 | 1.25E-06 | 0.00101 |
| PLAAT2 | 2.107874 | 2.87E-06 | 0.001546 |
| REG1B | 2.075396 | 1.42E-06 | 0.00101 |
| GUCA2B | 2.005068 | 1.47E-05 | 0.00521 |
| AQP9 | 1.884146 | 3.93E-06 | 0.001819 |
| BTNL8 | 1.876521 | 2.20E-06 | 0.001325 |
| AKR1B10 | 1.814014 | 1.84E-06 | 0.001233 |
| TNFAIP6 | 1.813296 | 5.73E-05 | 0.015351 |
| EMP1 | 1.788405 | 2.07E-06 | 0.001315 |
| AXDND1 | 1.732547 | 5.23E-06 | 0.002174 |
| DMBT1 | 1.708362 | 6.00E-05 | 0.01573 |
| ABHD11-AS1 | 1.704457 | 1.10E-05 | 0.004039 |
| HLA-DRB6 | 1.685328 | 4.90E-05 | 0.01408 |
| IL1RN | 1.653195 | 4.07E-06 | 0.001819 |
| CXCL8 | 1.613696 | 3.28E-05 | 0.010394 |
| AC009088.4 | 1.611773 | 7.21E-05 | 0.017733 |
| IL1B | 1.567226 | 1.11E-05 | 0.004039 |
| P3H2 | 1.538631 | 3.91E-05 | 0.01164 |
| SULT1A2 | 1.498574 | 0.000122 | 0.028799 |
| F2RL1 | 1.495586 | 5.06E-05 | 0.014189 |
| TRIM36 | 1.494422 | 0.000199 | 0.043702 |
| UGT2B17 | 1.487079 | 5.43E-05 | 0.014865 |
| ARL14 | 1.481388 | 3.77E-05 | 0.01164 |
| S100A9 | 1.466568 | 6.36E-05 | 0.016324 |
| CA2 | 1.460485 | 2.72E-05 | 0.00886 |
| PLAUR | 1.434803 | 0.000253 | 0.04844 |
| NDUFB3 | 1.424068 | 0.000218 | 0.045982 |
| HBA1 | 1.389576 | 0.000283 | 0.05165 |
| CDA | 1.385897 | 0.000253 | 0.04844 |
| TSPAN15 | 1.367116 | 9.70E-05 | 0.023389 |
| SLC26A3 | 1.348756 | 0.00015 | 0.034797 |
| GUCA2A | 1.333416 | 0.000266 | 0.049273 |
| SULT1A1 | 1.297644 | 0.00022 | 0.045982 |
| GPA33 | 1.292808 | 0.000258 | 0.048584 |
| NAPRT | 1.285522 | 0.000165 | 0.037496 |
| HBA2 | 1.279113 | 0.000231 | 0.047197 |
| CGN | 1.266571 | 0.000251 | 0.04844 |
| IGHG4 | -1.2764 | 0.000221 | 0.045982 |
| IGHG3 | -1.35564 | 6.73E-05 | 0.016904 |
| GREM2 | -1.39158 | 0.000191 | 0.042747 |
| IGHGP | -1.42799 | 3.96E-05 | 0.01164 |
| INSL5 | -1.48576 | 0.000246 | 0.04844 |
| ABI3BP | -1.5493 | 1.99E-05 | 0.006867 |
| CCL19 | -1.56637 | 2.37E-05 | 0.007943 |
| AC073140.1 | -1.97043 | 1.62E-08 | 2.61E-05 |
| AC119673.2 | -2.13058 | 2.95E-06 | 0.001546 |
| NTRK2 | -2.14208 | 5.01E-06 | 0.002156 |
| DEFB4B | -2.3487 | 7.40E-06 | 0.002876 |
| DEFB4A | -2.36247 | 6.60E-06 | 0.002654 |
| AC108865.2 | -2.86163 | 1.42E-06 | 0.00101 |
| HOXD11 | -2.93382 | 1.68E-08 | 2.61E-05 |

**Supplementary table 12**

**Differentially expressed genes in the colonic mucosa between responders and non-responders.**

| **Genes** | **logFC** | **PValue** | **FDR** |
| --- | --- | --- | --- |
| REG1B | -6.21592 | 1.49E-66 | 1.78E-62 |
| REG3A | -5.35168 | 5.66E-54 | 3.39E-50 |
| PRSS2 | -4.82019 | 1.15E-42 | 2.28E-39 |
| DEFA5 | -4.72246 | 3.08E-44 | 1.23E-40 |
| DEFA6 | -4.68799 | 6.50E-43 | 1.55E-39 |
| REG1A | -4.67854 | 1.08E-43 | 3.22E-40 |
| HCAR3 | -4.2837 | 1.45E-26 | 2.48E-23 |
| PROK2 | -4.00638 | 1.40E-19 | 1.67E-16 |
| TREM1 | -3.53018 | 1.61E-14 | 8.36E-12 |
| CXCR2 | -3.46139 | 4.38E-19 | 4.76E-16 |
| SPP1 | -3.39712 | 1.28E-18 | 1.18E-15 |
| CXCR1 | -3.31332 | 4.56E-13 | 1.88E-10 |
| HCAR2 | -3.258 | 8.09E-17 | 6.45E-14 |
| AQP9 | -3.23002 | 1.05E-16 | 7.88E-14 |
| FCGR3B | -3.18213 | 5.92E-19 | 5.90E-16 |
| S100A8 | -3.15651 | 1.04E-20 | 1.49E-17 |
| S100A9 | -3.10522 | 1.12E-20 | 1.49E-17 |
| FPR1 | -3.03815 | 5.46E-16 | 3.84E-13 |
| CXCL8 | -2.8899 | 2.44E-15 | 1.33E-12 |
| G0S2 | -2.82144 | 6.29E-16 | 4.18E-13 |
| CHI3L1 | -2.74476 | 1.68E-15 | 9.56E-13 |
| MMP1 | -2.73733 | 7.00E-16 | 4.40E-13 |
| CSF3R | -2.70345 | 9.46E-14 | 4.52E-11 |
| SERPINE1 | -2.70303 | 5.23E-12 | 2.01E-09 |
| MMP3 | -2.6434 | 1.06E-15 | 6.35E-13 |
| CLEC4E | -2.62466 | 1.53E-10 | 4.59E-08 |
| ITLN2 | -2.56541 | 1.79E-13 | 7.92E-11 |
| CXCL5 | -2.52957 | 5.60E-14 | 2.79E-11 |
| SIRPB1 | -2.52757 | 9.19E-10 | 2.44E-07 |
| VNN2 | -2.51684 | 3.09E-11 | 1.09E-08 |
| TNFAIP6 | -2.45611 | 1.95E-09 | 4.96E-07 |
| MMP9 | -2.38806 | 4.44E-13 | 1.88E-10 |
| IGHG1 | -2.38037 | 1.27E-13 | 5.86E-11 |
| SLC11A1 | -2.34146 | 3.61E-09 | 8.62E-07 |
| FCN1 | -2.32928 | 2.12E-08 | 4.37E-06 |
| INHBA | -2.31122 | 3.89E-12 | 1.55E-09 |
| HLA-DQA2 | -2.30983 | 8.35E-11 | 2.71E-08 |
| TCN1 | -2.3019 | 1.78E-10 | 5.19E-08 |
| IGHD | -2.23265 | 3.41E-10 | 9.43E-08 |
| MNDA | -2.22359 | 5.05E-11 | 1.73E-08 |
| SAA1 | -2.19946 | 2.23E-10 | 6.35E-08 |
| KRT7 | -2.19196 | 1.31E-08 | 2.86E-06 |
| PLEK | -2.18836 | 1.58E-11 | 5.74E-09 |
| IGHM | -2.17234 | 1.21E-11 | 4.52E-09 |
| IL11 | -2.1536 | 3.39E-07 | 5.17E-05 |
| IGHV3-15 | -2.11384 | 3.47E-10 | 9.43E-08 |
| KRT17 | -2.11208 | 7.84E-09 | 1.74E-06 |
| MMP10 | -2.07249 | 1.56E-08 | 3.27E-06 |
| IGHG3 | -2.05085 | 1.51E-10 | 4.59E-08 |
| SELL | -1.97523 | 2.82E-09 | 7.02E-07 |
| FFAR2 | -1.96908 | 4.94E-08 | 9.37E-06 |
| TRIM40 | -1.96608 | 3.60E-08 | 6.94E-06 |
| GNLY | -1.95317 | 2.33E-07 | 3.77E-05 |
| IGHG2 | -1.95182 | 1.20E-09 | 3.12E-07 |
| PLAU | -1.94491 | 3.02E-07 | 4.81E-05 |
| CXCL11 | -1.9407 | 5.24E-08 | 9.78E-06 |
| GREM1 | -1.93984 | 3.14E-09 | 7.66E-07 |
| BCL2A1 | -1.91019 | 2.53E-08 | 5.13E-06 |
| IL1RN | -1.8985 | 3.85E-09 | 9.02E-07 |
| CXCL9 | -1.89736 | 1.35E-08 | 2.87E-06 |
| IGHGP | -1.87509 | 5.47E-09 | 1.26E-06 |
| TWIST1 | -1.8745 | 3.03E-06 | 0.000356 |
| LILRB3 | -1.87359 | 3.11E-07 | 4.82E-05 |
| FCGR3A | -1.85853 | 2.22E-07 | 3.63E-05 |
| IGHV3-30 | -1.85171 | 3.42E-07 | 5.17E-05 |
| CHST2 | -1.84938 | 5.01E-07 | 7.30E-05 |
| TNIP3 | -1.83282 | 3.14E-08 | 6.16E-06 |
| CCL3 | -1.82928 | 2.01E-07 | 3.34E-05 |
| IL1B | -1.81916 | 3.14E-08 | 6.16E-06 |
| CD79B | -1.81913 | 3.11E-07 | 4.82E-05 |
| PTGDS | -1.81 | 5.97E-08 | 1.08E-05 |
| TAGAP | -1.80984 | 5.21E-07 | 7.51E-05 |
| NCF2 | -1.79047 | 1.29E-07 | 2.21E-05 |
| CR1 | -1.77468 | 4.72E-06 | 0.000517 |
| TCL1A | -1.73989 | 3.00E-06 | 0.000355 |
| NNMT | -1.73633 | 4.41E-07 | 6.60E-05 |
| PDPN | -1.71866 | 1.53E-06 | 0.000201 |
| C5AR1 | -1.7153 | 1.94E-06 | 0.000252 |
| IGHG4 | -1.71144 | 9.27E-08 | 1.65E-05 |
| ICAM1 | -1.70946 | 5.66E-07 | 7.96E-05 |
| LRRK2 | -1.70503 | 2.59E-06 | 0.000324 |
| FCGR2A | -1.6993 | 7.09E-07 | 9.85E-05 |
| DES | -1.69553 | 5.30E-07 | 7.54E-05 |
| IGLC5 | -1.69489 | 1.22E-06 | 0.000161 |
| BATF | -1.69414 | 2.13E-05 | 0.001834 |
| FGR | -1.69378 | 3.98E-06 | 0.000445 |
| CLDN2 | -1.67999 | 7.99E-07 | 0.000109 |
| MADCAM1 | -1.67391 | 2.20E-06 | 0.00028 |
| S1PR4 | -1.67247 | 2.15E-05 | 0.00184 |
| C4A | -1.66271 | 7.64E-06 | 0.000754 |
| C4B | -1.66271 | 7.64E-06 | 0.000754 |
| CXCL10 | -1.63353 | 6.22E-06 | 0.00063 |
| C3 | -1.62272 | 9.63E-07 | 0.000129 |
| STEAP4 | -1.62006 | 7.86E-05 | 0.005398 |
| SELP | -1.61978 | 1.84E-05 | 0.001606 |
| BGN | -1.61785 | 2.95E-06 | 0.000352 |
| NCF1C | -1.61751 | 3.62E-06 | 0.000416 |
| SOCS1 | -1.61201 | 2.91E-05 | 0.002379 |
| MS4A1 | -1.59759 | 8.03E-07 | 0.000109 |
| CSGALNACT1 | -1.59041 | 3.20E-05 | 0.002567 |
| CR2 | -1.58736 | 2.61E-06 | 0.000324 |
| NFAM1 | -1.58219 | 7.02E-05 | 0.004878 |
| FADS2 | -1.55551 | 1.10E-05 | 0.001007 |
| LPL | -1.54703 | 3.19E-05 | 0.002567 |
| MROH6 | -1.54537 | 9.76E-06 | 0.000926 |
| SOCS3 | -1.54008 | 2.67E-06 | 0.000325 |
| GBP5 | -1.53519 | 8.12E-06 | 0.000795 |
| LUCAT1 | -1.53249 | 1.40E-05 | 0.001242 |
| CCL19 | -1.53199 | 3.68E-06 | 0.000419 |
| MYO1F | -1.51884 | 1.05E-05 | 0.000977 |
| BASP1 | -1.51389 | 3.54E-06 | 0.000411 |
| SPI1 | -1.51243 | 2.95E-05 | 0.002395 |
| PLA2G2A | -1.50443 | 2.77E-06 | 0.000334 |
| SNX10 | -1.49632 | 1.18E-05 | 0.001065 |
| SCN1B | -1.4932 | 6.25E-05 | 0.004446 |
| KYNU | -1.49299 | 4.49E-05 | 0.003394 |
| CLU | -1.48956 | 3.95E-06 | 0.000445 |
| TNS4 | -1.48326 | 2.42E-05 | 0.002023 |
| ALDH1A2 | -1.48103 | 0.000126 | 0.008292 |
| AIM2 | -1.48057 | 5.73E-05 | 0.004178 |
| CCL4 | -1.47757 | 1.99E-05 | 0.001726 |
| CHI3L2 | -1.46785 | 0.00013 | 0.008515 |
| IGLC1 | -1.46738 | 4.87E-06 | 0.00053 |
| KLK10 | -1.46433 | 1.51E-05 | 0.001324 |
| DMBT1 | -1.45929 | 5.76E-06 | 0.000605 |
| IGLC2 | -1.45616 | 5.66E-06 | 0.000604 |
| IGLL5 | -1.45548 | 5.82E-06 | 0.000605 |
| PI3 | -1.45328 | 6.10E-06 | 0.000623 |
| IGHV3-7 | -1.45322 | 3.93E-05 | 0.003034 |
| HLA-DRB6 | -1.45257 | 1.05E-05 | 0.000977 |
| PLA2G7 | -1.45161 | 2.24E-05 | 0.001902 |
| LTA | -1.45049 | 0.000148 | 0.009324 |
| CXCL13 | -1.44442 | 1.22E-05 | 0.001084 |
| FCRLA | -1.44037 | 0.000125 | 0.00829 |
| CXCR5 | -1.43709 | 0.000262 | 0.014438 |
| PDE4B | -1.43022 | 2.54E-05 | 0.002111 |
| DOK3 | -1.42241 | 0.000128 | 0.008376 |
| IGLC3 | -1.41973 | 9.72E-06 | 0.000926 |
| IDO1 | -1.41963 | 7.35E-05 | 0.005077 |
| IGLC6 | -1.41395 | 0.000104 | 0.006995 |
| FOSL1 | -1.41067 | 0.000509 | 0.024323 |
| LST1 | -1.41015 | 0.000148 | 0.009324 |
| LILRB2 | -1.40244 | 7.00E-05 | 0.004878 |
| AL353804.4 | -1.39076 | 0.000219 | 0.012652 |
| VWF | -1.3878 | 6.70E-05 | 0.00471 |
| IGHV3-33 | -1.38718 | 0.000256 | 0.014157 |
| CCN1 | -1.3843 | 6.18E-05 | 0.004425 |
| LPCAT1 | -1.37091 | 5.00E-05 | 0.003754 |
| RASGRP1 | -1.37068 | 0.00017 | 0.010468 |
| FCER1G | -1.36826 | 4.38E-05 | 0.003337 |
| NCF1B | -1.36749 | 0.000214 | 0.012472 |
| RIPOR2 | -1.36741 | 3.84E-05 | 0.002983 |
| CFI | -1.36641 | 6.29E-05 | 0.004446 |
| TYMP | -1.35796 | 3.40E-05 | 0.002692 |
| CD180 | -1.35695 | 0.000255 | 0.014157 |
| MMP12 | -1.35465 | 3.74E-05 | 0.002923 |
| SRGN | -1.34576 | 3.25E-05 | 0.002593 |
| MME | -1.34397 | 0.00025 | 0.013973 |
| MZB1 | -1.34164 | 5.03E-05 | 0.003754 |
| HAPLN3 | -1.3407 | 0.000791 | 0.034251 |
| RSPO3 | -1.33938 | 0.000179 | 0.010876 |
| TIGIT | -1.33842 | 0.000327 | 0.017239 |
| TRAF1 | -1.33689 | 0.000354 | 0.018026 |
| ITGAX | -1.33093 | 0.000225 | 0.012797 |
| EMP3 | -1.32946 | 0.000241 | 0.01357 |
| PCED1B-AS1 | -1.32333 | 0.000186 | 0.011111 |
| CD79A | -1.32088 | 5.96E-05 | 0.00429 |
| NOS2 | -1.31761 | 5.87E-05 | 0.004256 |
| MOXD1 | -1.31729 | 0.000761 | 0.033439 |
| VPREB3 | -1.31188 | 0.000308 | 0.016462 |
| MRAS | -1.31137 | 0.000734 | 0.0325 |
| SLCO1B3 | -1.3097 | 0.000302 | 0.016266 |
| DYSF | -1.30499 | 0.000343 | 0.01767 |
| TM4SF20 | -1.30254 | 0.000175 | 0.010703 |
| PAX5 | -1.30196 | 0.000107 | 0.00717 |
| CREB5 | -1.30095 | 0.000643 | 0.029244 |
| CD40 | -1.30084 | 0.000207 | 0.012227 |
| LTBP2 | -1.29213 | 0.000774 | 0.033888 |
| CHST11 | -1.28829 | 0.000333 | 0.017352 |
| SLC7A5 | -1.28263 | 0.000297 | 0.016082 |
| SEMA4A | -1.27965 | 0.000589 | 0.027196 |
| CCL18 | -1.27963 | 0.000214 | 0.012472 |
| COL7A1 | -1.27599 | 0.000608 | 0.02794 |
| TNC | -1.26819 | 0.000266 | 0.014579 |
| COL8A1 | -1.26723 | 0.000722 | 0.032196 |
| LCN2 | -1.26431 | 8.78E-05 | 0.005997 |
| GZMA | -1.26413 | 0.000297 | 0.016082 |
| CD19 | -1.26402 | 0.000362 | 0.018322 |
| CD22 | -1.25309 | 0.000309 | 0.016462 |
| CCL21 | -1.25299 | 0.000183 | 0.011011 |
| GNA15 | -1.25223 | 0.000372 | 0.018771 |
| DIO2 | -1.25085 | 0.000464 | 0.022811 |
| CXCL1 | -1.24716 | 0.000136 | 0.008775 |
| KLK7 | -1.246 | 0.001109 | 0.044624 |
| KLHL6 | -1.2453 | 0.000226 | 0.012797 |
| P2RY13 | -1.24481 | 0.000499 | 0.024141 |
| EIF1AD | -1.24444 | 0.001346 | 0.05127 |
| DTX1 | -1.24267 | 0.001244 | 0.048286 |
| PTGS2 | -1.2401 | 0.000167 | 0.010398 |
| IGSF6 | -1.22952 | 0.000226 | 0.012797 |
| GNB5 | -1.22879 | 0.00034 | 0.017618 |
| ADGRE2 | -1.22296 | 0.000542 | 0.025296 |
| RASD2 | -1.22178 | 0.000393 | 0.019657 |
| CXCL2 | -1.22082 | 0.000469 | 0.022899 |
| CD7 | -1.22027 | 0.000879 | 0.037393 |
| SELPLG | -1.22014 | 0.000852 | 0.036631 |
| NCF1 | -1.21953 | 0.000874 | 0.037312 |
| PTPRC | -1.2186 | 0.00017 | 0.010468 |
| WARS | -1.21846 | 0.000181 | 0.010897 |
| HLA-DOB | -1.2174 | 0.001211 | 0.047306 |
| HLA-DRB5 | -1.21609 | 0.000177 | 0.010785 |
| MMP7 | -1.21338 | 0.000455 | 0.022465 |
| LTF | -1.2132 | 0.00065 | 0.029413 |
| IL4I1 | -1.21112 | 0.000891 | 0.037632 |
| ACTG2 | -1.2106 | 0.000348 | 0.017801 |
| AGT | -1.20867 | 0.001144 | 0.045456 |
| RAB31 | -1.20849 | 0.000217 | 0.012573 |
| IRF8 | -1.20578 | 0.000758 | 0.033432 |
| RGS2 | -1.20221 | 0.000246 | 0.01379 |
| CD55 | -1.2013 | 0.000204 | 0.012107 |
| TMEM154 | -1.19945 | 0.000702 | 0.031541 |
| PPP4C | -1.19556 | 0.00091 | 0.038017 |
| ZBP1 | -1.19335 | 0.001144 | 0.045456 |
| PRELP | -1.18471 | 0.000884 | 0.037449 |
| PLEKHB1 | -1.18038 | 0.001269 | 0.048914 |
| KLHL5 | -1.18001 | 0.000622 | 0.028498 |
| RFTN1 | -1.17517 | 0.000374 | 0.018783 |
| NIBAN1 | -1.16905 | 0.000404 | 0.020143 |
| FDCSP | -1.16506 | 0.000344 | 0.01767 |
| DERL3 | -1.16458 | 0.000429 | 0.021272 |
| SLC2A3 | -1.15801 | 0.000514 | 0.024375 |
| EPHA2 | -1.15601 | 0.001305 | 0.04999 |
| CXCR4 | -1.15365 | 0.000578 | 0.026896 |
| BANK1 | -1.15364 | 0.000714 | 0.031981 |
| PTPRCAP | -1.15217 | 0.001298 | 0.049874 |
| CD37 | -1.14451 | 0.000485 | 0.023564 |
| IKZF3 | -1.14426 | 0.000514 | 0.024375 |
| KCNN3 | -1.14012 | 0.001421 | 0.053762 |
| ARID5A | -1.13624 | 0.001155 | 0.045572 |
| TIMP1 | -1.13259 | 0.000501 | 0.024159 |
| TRIB2 | -1.13019 | 0.000802 | 0.034613 |
| CHGB | -1.12941 | 0.000788 | 0.034251 |
| MGP | -1.12857 | 0.000962 | 0.039535 |
| DUOX2 | -1.12811 | 0.000469 | 0.022899 |
| SULF1 | -1.12696 | 0.000951 | 0.039326 |
| IGFBP5 | -1.12476 | 0.000537 | 0.025178 |
| CD53 | -1.12473 | 0.00098 | 0.039986 |
| POU2AF1 | -1.1242 | 0.000951 | 0.039326 |
| FCMR | -1.11823 | 0.001132 | 0.045407 |
| LYZ | -1.11302 | 0.000585 | 0.027122 |
| GMFG | -1.10887 | 0.001262 | 0.048815 |
| PCSK1 | -1.10787 | 0.001012 | 0.041088 |
| PPP1R18 | -1.10672 | 0.000788 | 0.034251 |
| LCP1 | -1.10663 | 0.000687 | 0.030978 |
| FADS1 | -1.10287 | 0.001371 | 0.052039 |
| THY1 | -1.09774 | 0.000972 | 0.039807 |
| SLA | -1.08467 | 0.001187 | 0.046653 |
| POU2F2 | -1.07985 | 0.001014 | 0.041088 |
| APIP | -1.07874 | 0.001055 | 0.042587 |
| IFITM2 | -1.07603 | 0.000901 | 0.037796 |
| CXCL3 | -1.06622 | 0.001145 | 0.045456 |
| UBD | -1.06353 | 0.00115 | 0.0455 |
| FOS | -1.04996 | 0.001217 | 0.047388 |
| CA1 | 1.132315 | 0.001347 | 0.05127 |
| PRSS23 | 1.188679 | 0.000858 | 0.03676 |
| GDF15 | 1.201306 | 0.000731 | 0.0325 |
| USP9Y | 1.240469 | 0.000896 | 0.037727 |
| LINC00668 | 1.241467 | 0.000935 | 0.038936 |
| MT1G | 1.274707 | 0.000334 | 0.017352 |
| RPS4Y1 | 1.282156 | 0.000333 | 0.017352 |
| SCGB2A1 | 1.301871 | 0.001206 | 0.047271 |
| GPR161 | 1.312336 | 0.000642 | 0.029244 |
| BMP3 | 1.350646 | 0.000518 | 0.024478 |
| MT1F | 1.385245 | 0.000146 | 0.009324 |
| SLC3A1 | 1.394127 | 0.000528 | 0.024845 |
| PTGDR | 1.395232 | 0.000507 | 0.024323 |
| NAALADL1 | 1.404421 | 0.000954 | 0.039326 |
| CDHR1 | 1.436668 | 0.000134 | 0.008675 |
| BEST2 | 1.437663 | 0.000112 | 0.007464 |
| PDE6A | 1.452754 | 0.000324 | 0.017132 |
| ABCG2 | 1.469079 | 5.52E-05 | 0.004049 |
| MT1H | 1.476573 | 0.000103 | 0.006976 |
| PRKY | 1.513812 | 0.000282 | 0.015376 |
| SLC26A2 | 1.515178 | 2.35E-05 | 0.001981 |
| UGT1A1 | 1.525212 | 0.000143 | 0.009167 |
| SYT17 | 1.542633 | 0.000225 | 0.012797 |
| LGALS2 | 1.561218 | 2.58E-05 | 0.002128 |
| AC087379.1 | 1.575354 | 0.00016 | 0.010018 |
| NTRK2 | 1.598279 | 0.00021 | 0.012359 |
| AC092902.4 | 1.617332 | 0.000324 | 0.017132 |
| PADI2 | 1.620626 | 8.99E-06 | 0.000874 |
| ZG16 | 1.633409 | 5.92E-06 | 0.00061 |
| TNNC2 | 1.653274 | 5.08E-05 | 0.003771 |
| SLC15A1 | 1.693457 | 3.58E-05 | 0.002818 |
| TXLNGY | 1.726406 | 5.00E-06 | 0.000538 |
| MT1M | 1.734259 | 4.48E-06 | 0.000496 |
| PCK1 | 1.744174 | 2.01E-06 | 0.000258 |
| HHIP | 1.776421 | 1.09E-05 | 0.001006 |
| SLC13A2 | 1.80121 | 0.000147 | 0.009324 |
| ADH1C | 1.865761 | 4.65E-07 | 6.87E-05 |
| SCUBE2 | 1.910266 | 6.98E-06 | 0.000701 |
| HOXD10 | 1.949047 | 1.19E-05 | 0.001068 |
| SERPINB3 | 1.951106 | 4.32E-05 | 0.003312 |
| GBA3 | 1.962126 | 2.64E-06 | 0.000325 |
| AC010086.3 | 1.980146 | 5.14E-05 | 0.003793 |
| HOXD11 | 2.003512 | 9.84E-06 | 0.000926 |
| AQP8 | 2.004266 | 5.55E-08 | 1.02E-05 |
| PRORY | 2.181662 | 9.75E-06 | 0.000926 |
| CHP2 | 2.195139 | 6.48E-09 | 1.46E-06 |
| LINC00278 | 2.247265 | 5.82E-06 | 0.000605 |
| TM6SF2 | 2.337862 | 1.81E-07 | 3.04E-05 |
| ANPEP | 2.492665 | 8.86E-11 | 2.79E-08 |
| DIO3 | 2.522236 | 1.09E-07 | 1.92E-05 |
| DIO3OS | 2.694339 | 1.24E-07 | 2.14E-05 |
| TTTY14 | 3.305594 | 8.38E-11 | 2.71E-08 |
| SLC51A | 5.222827 | 2.15E-18 | 1.84E-15 |
