# Supplementary figures and images for: Mechanistic insights into fecal microbiota transplantation for the treatment of ulcerative colitis: analysis of the STOP-Colitis trial
Source: J Crohns Colitis. 2026 Jan 23;20(3):jjag006. doi: 10.1093/ecco-jcc/jjag006 (PMC13012878; doi:10.1093/ecco-jcc/jjag006)

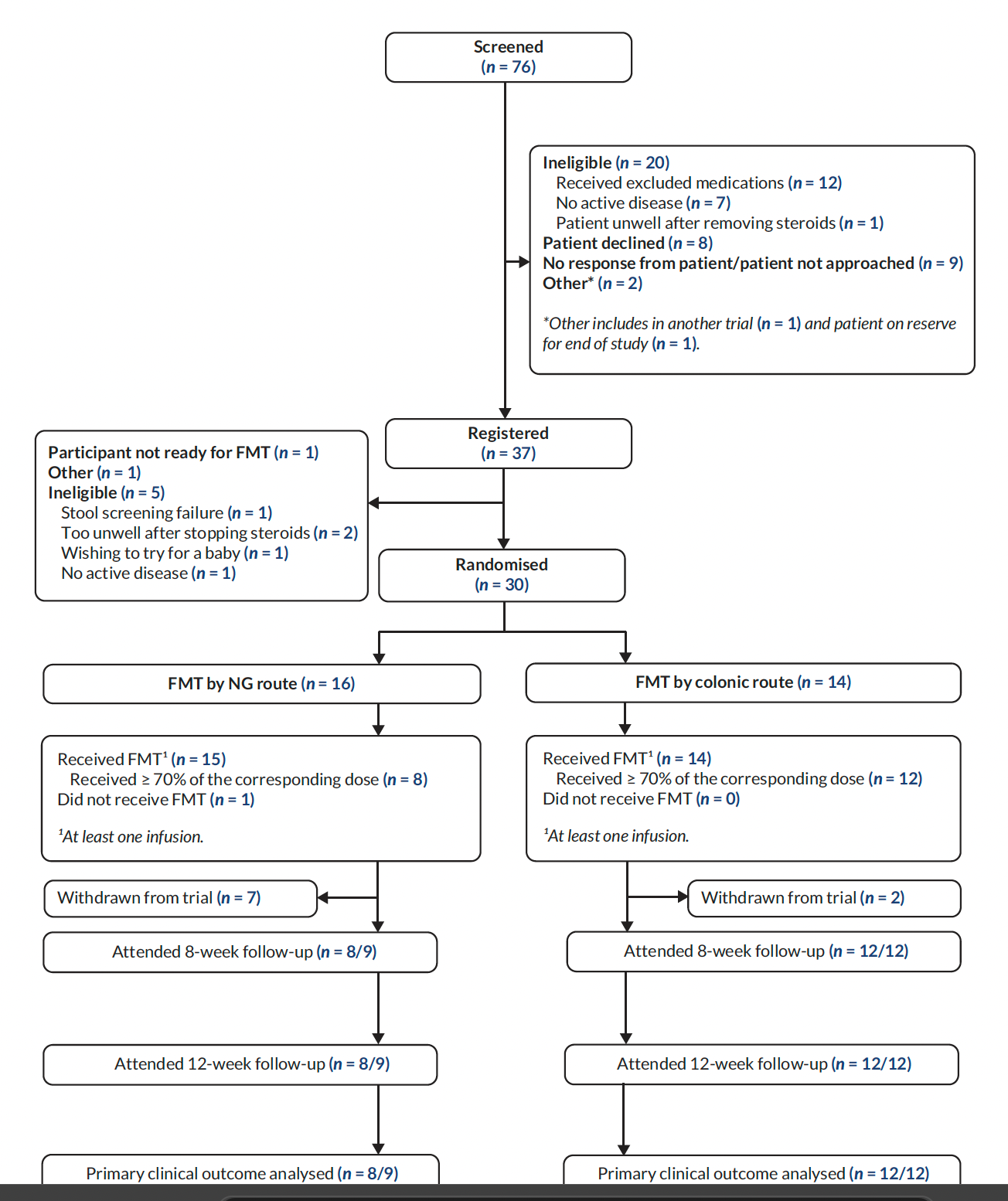

Supplement: jjag006_Supplementary_Data [file jjag006_supplementary_data.zip › Supplementary Figure 1 - Consort Flow Diagram.png]
